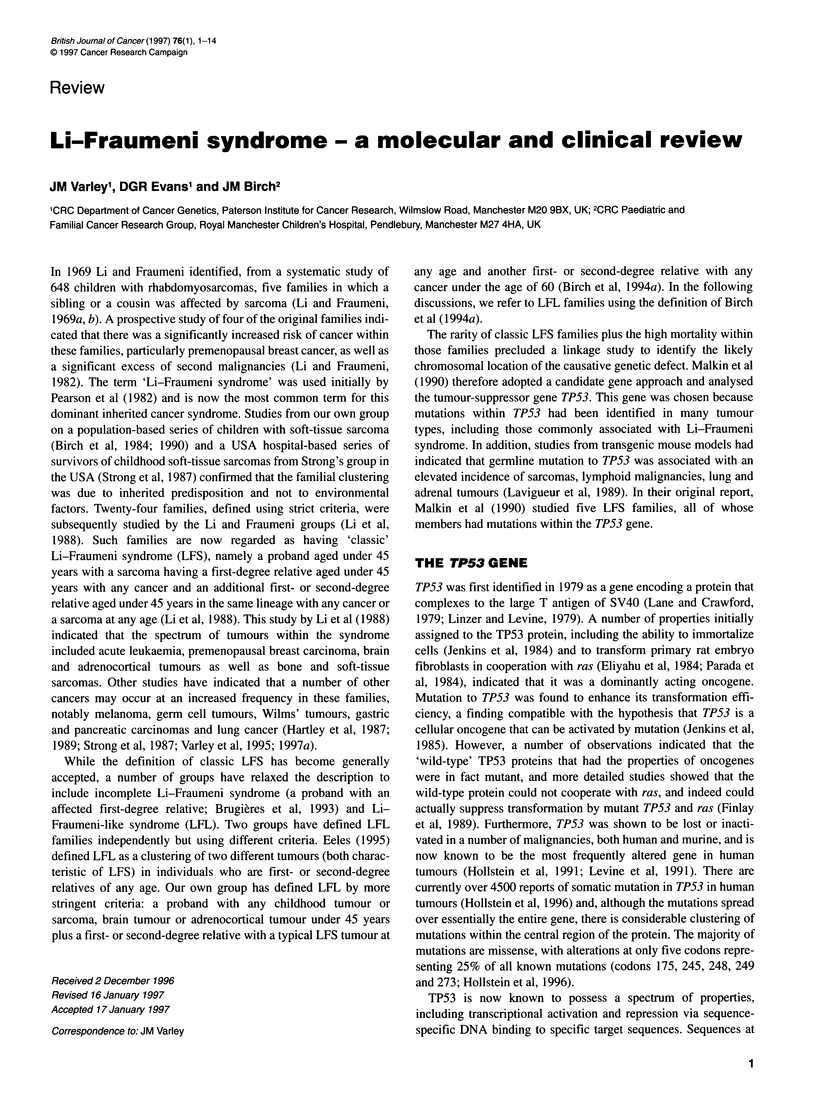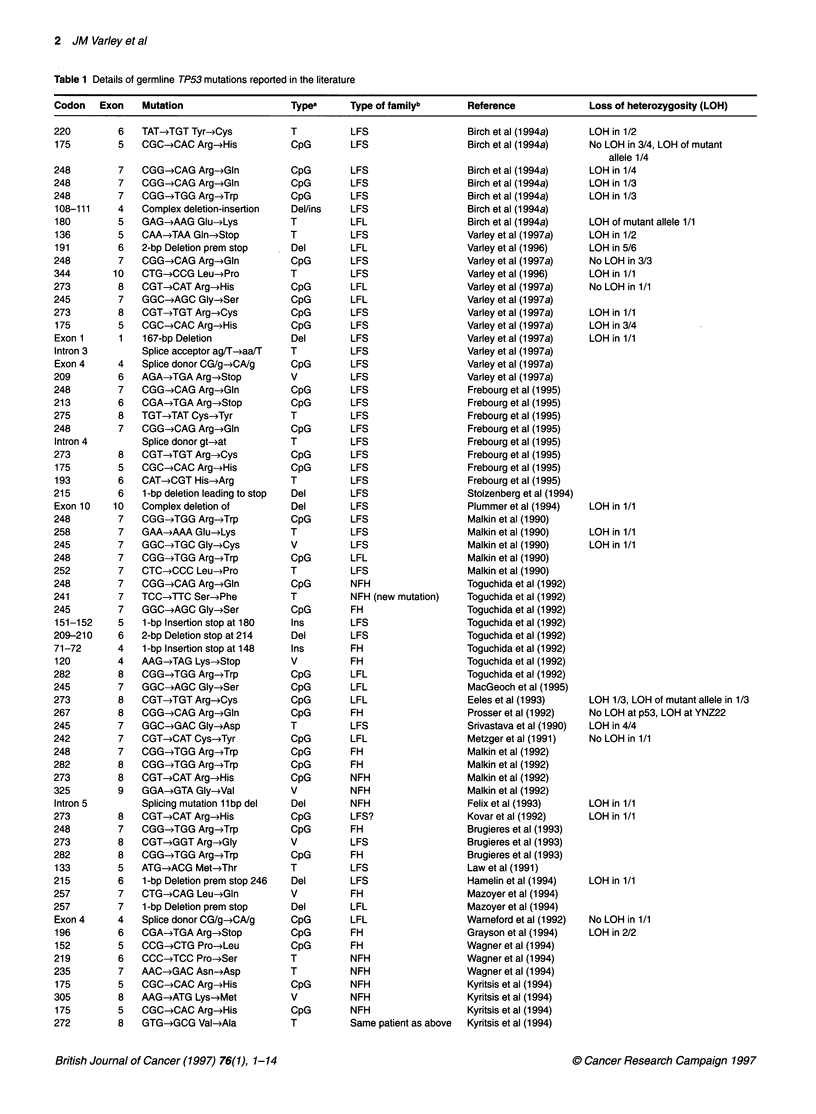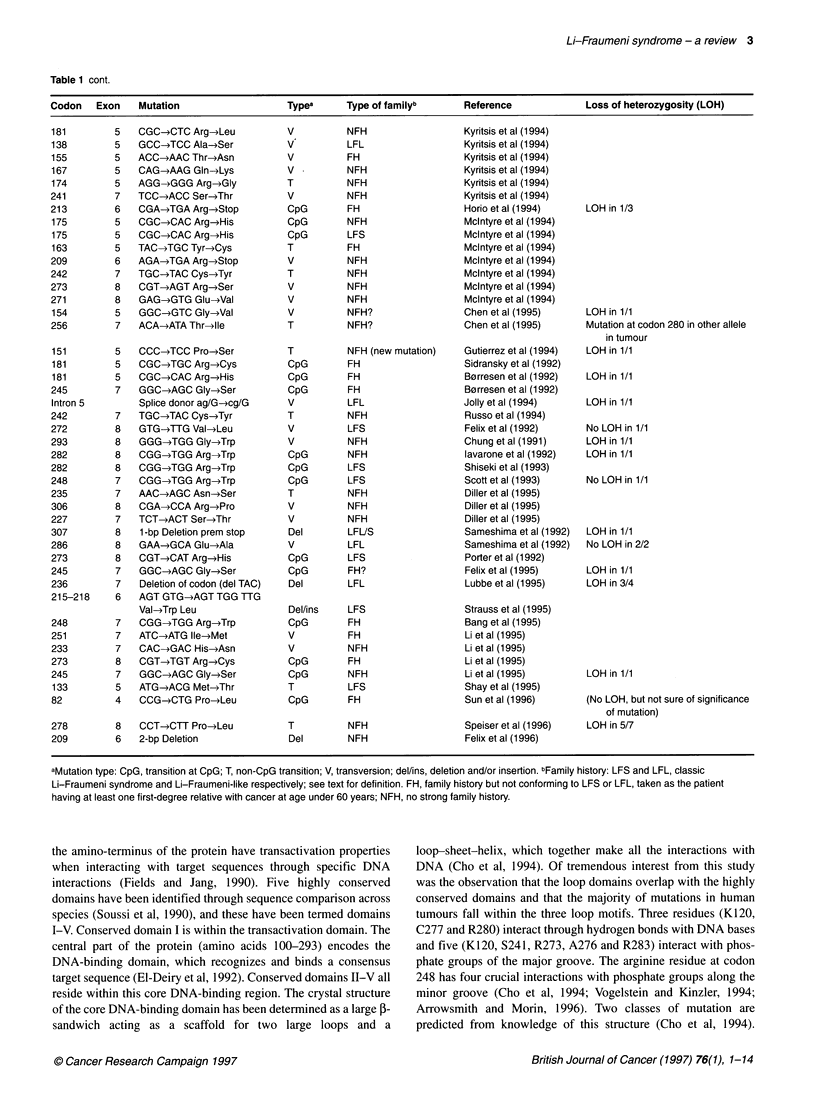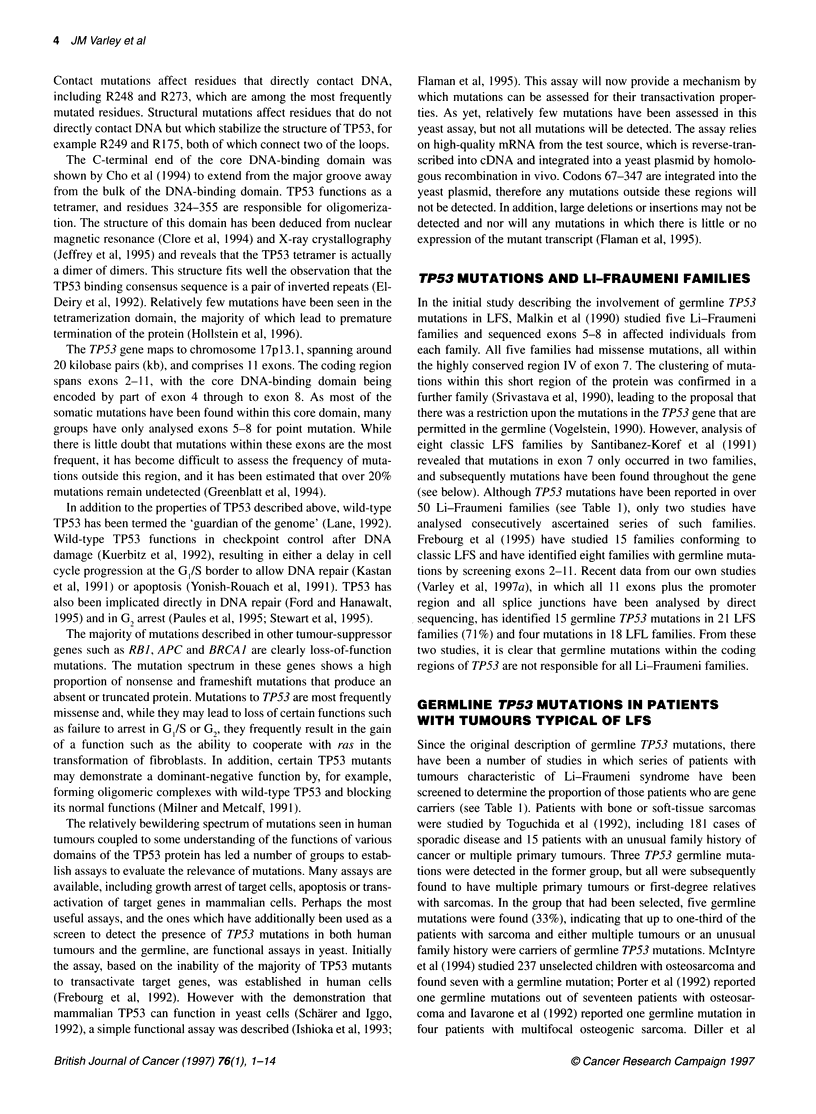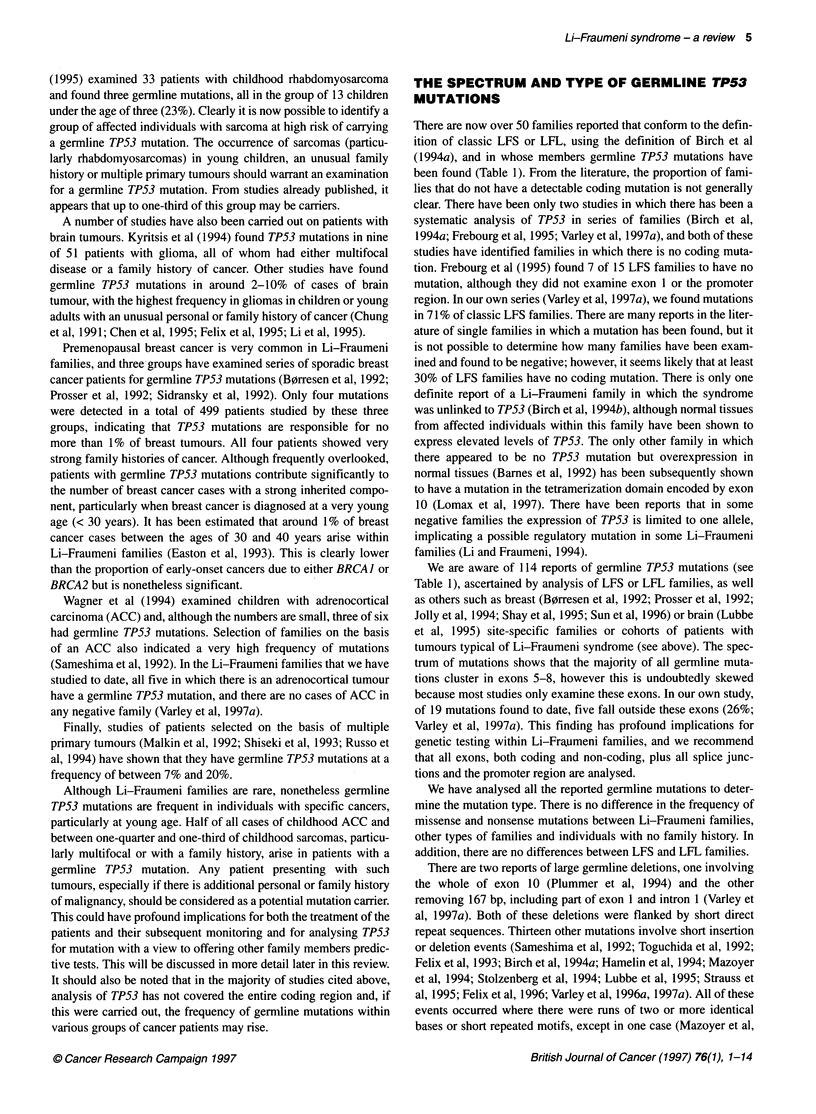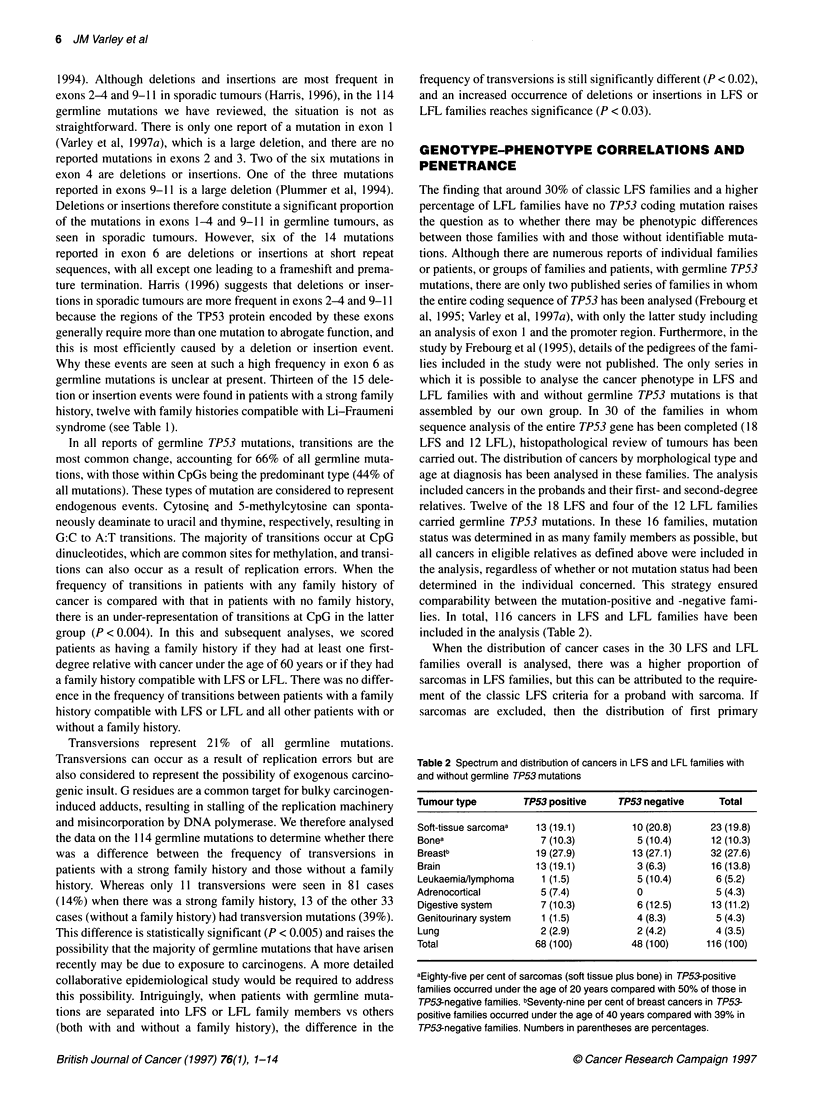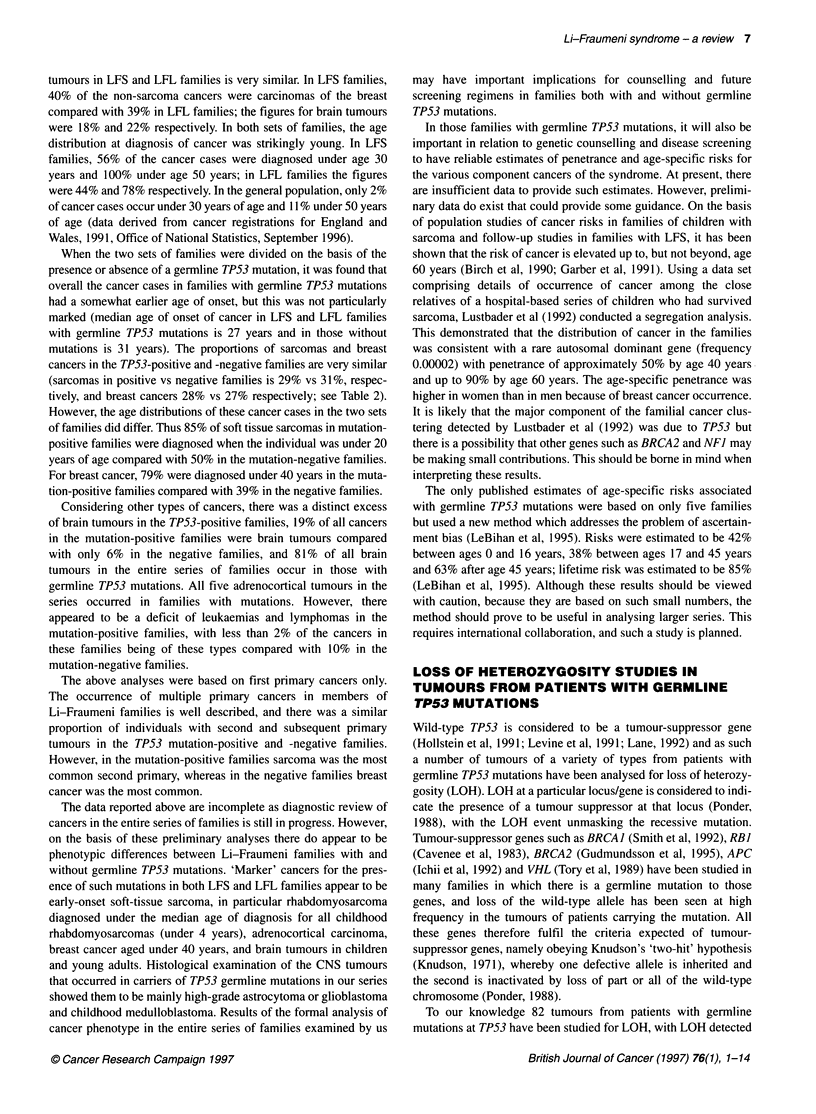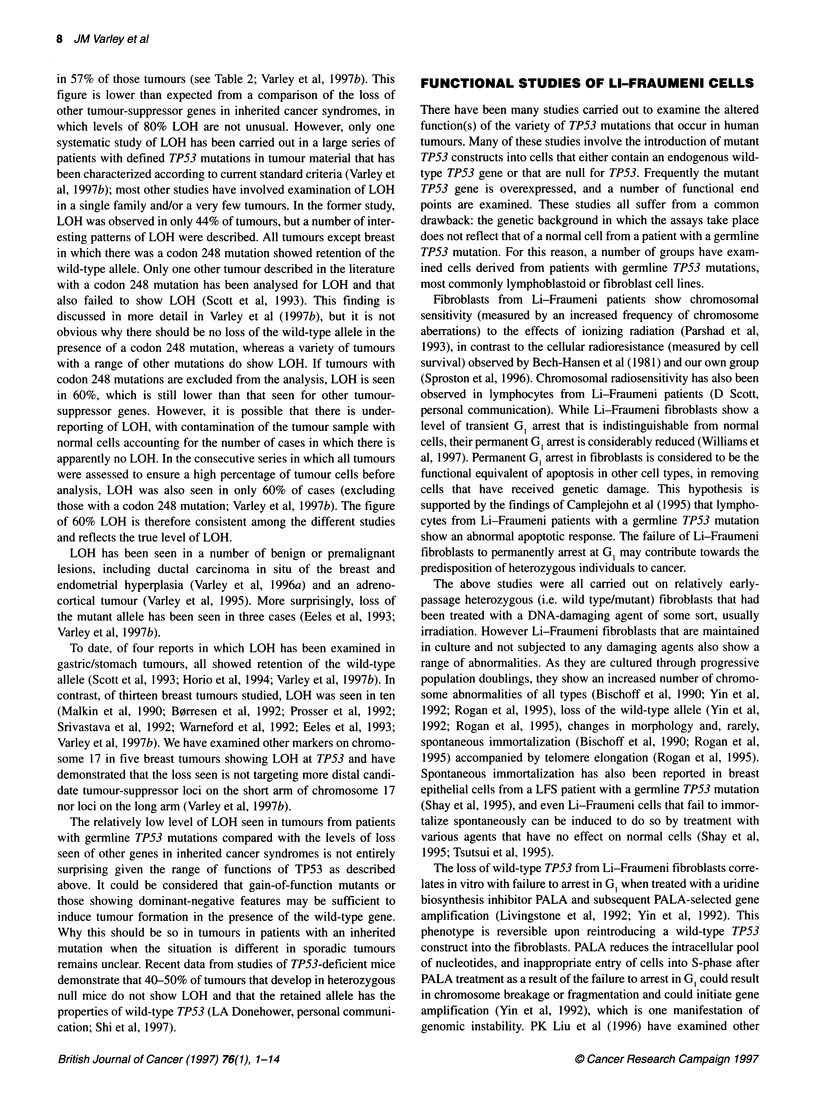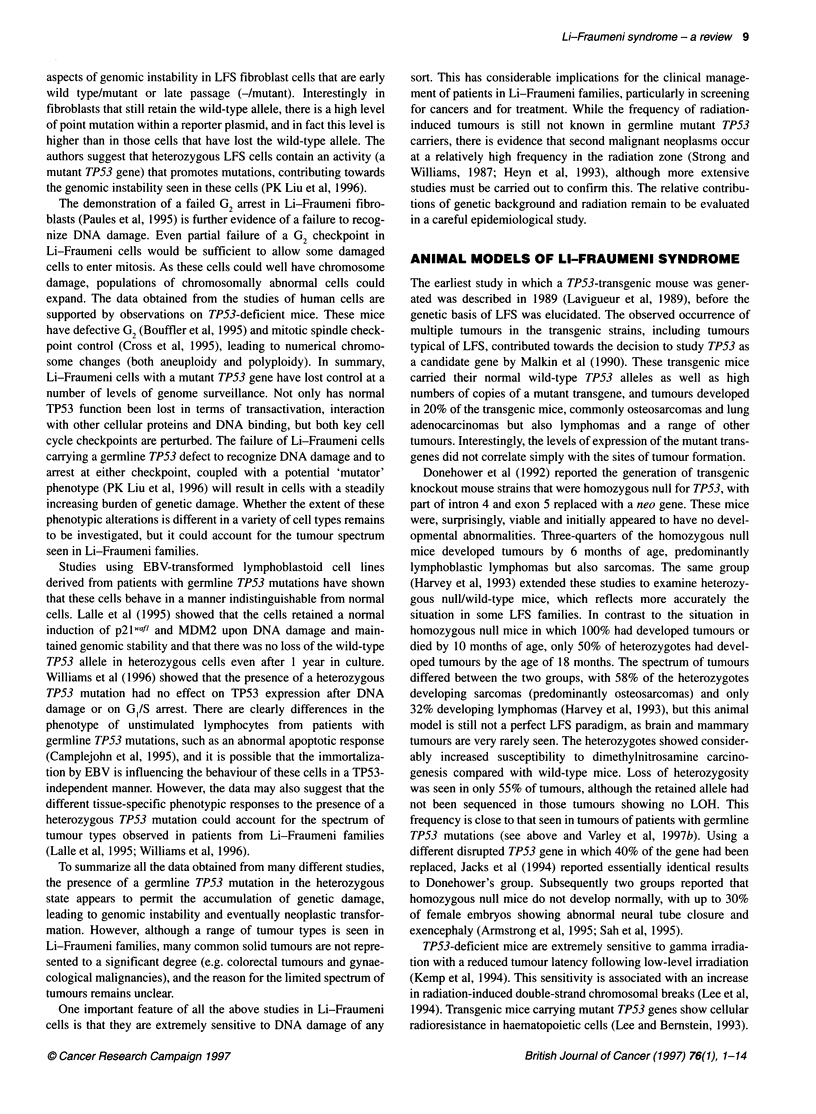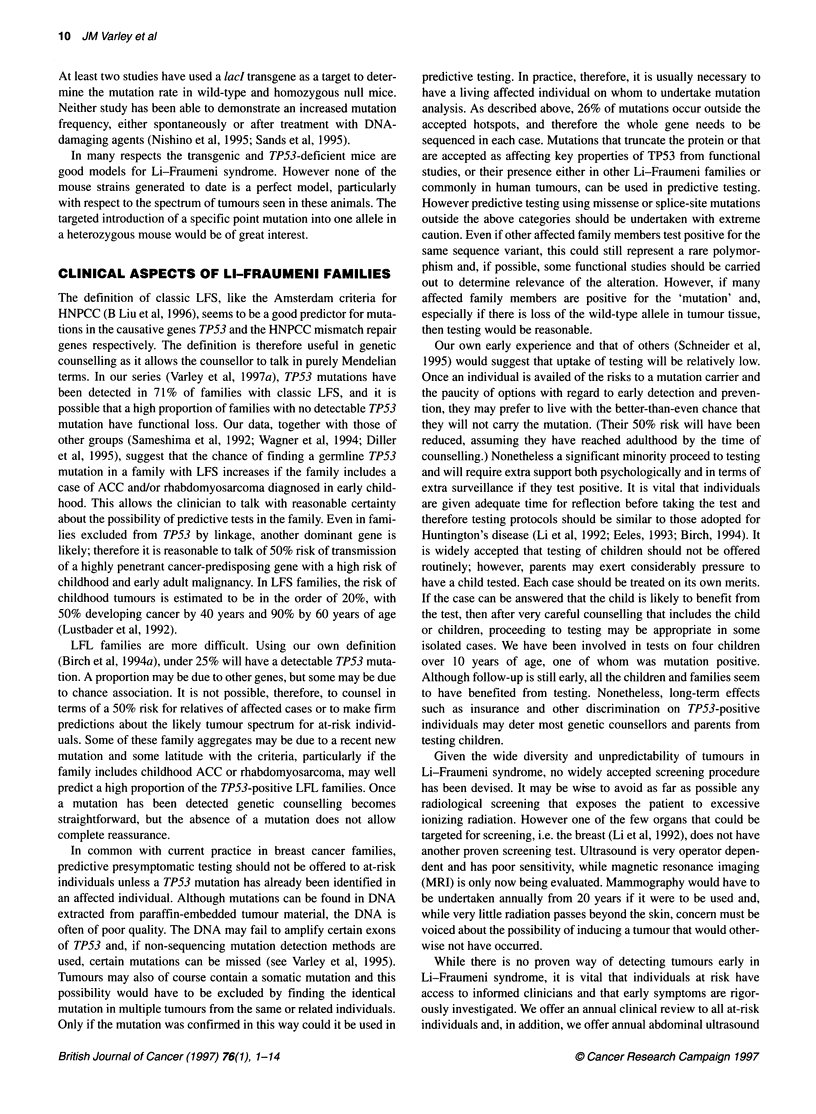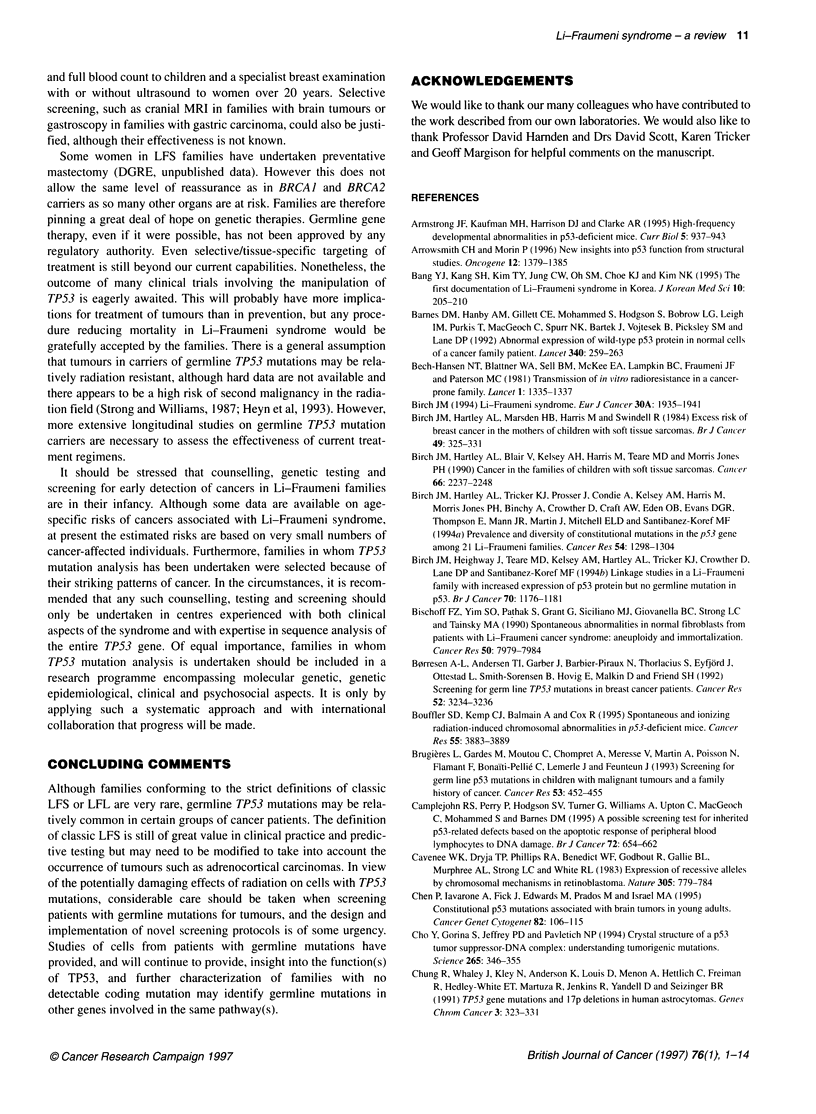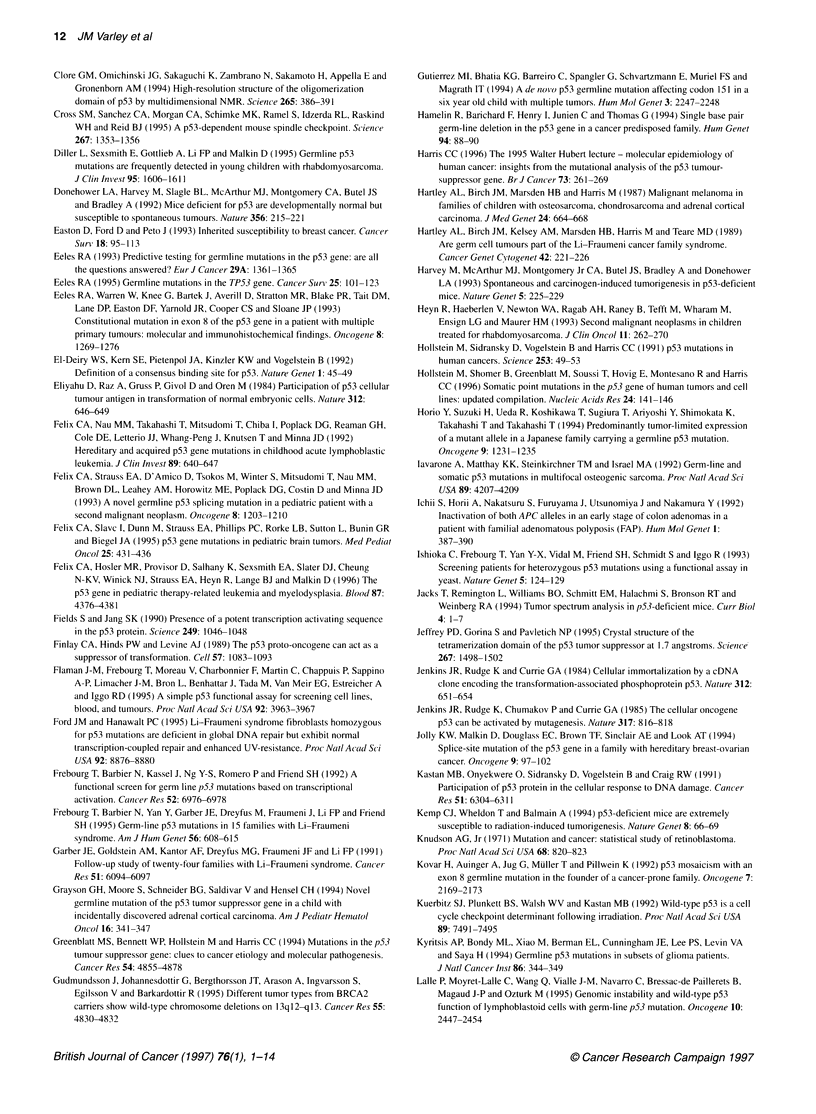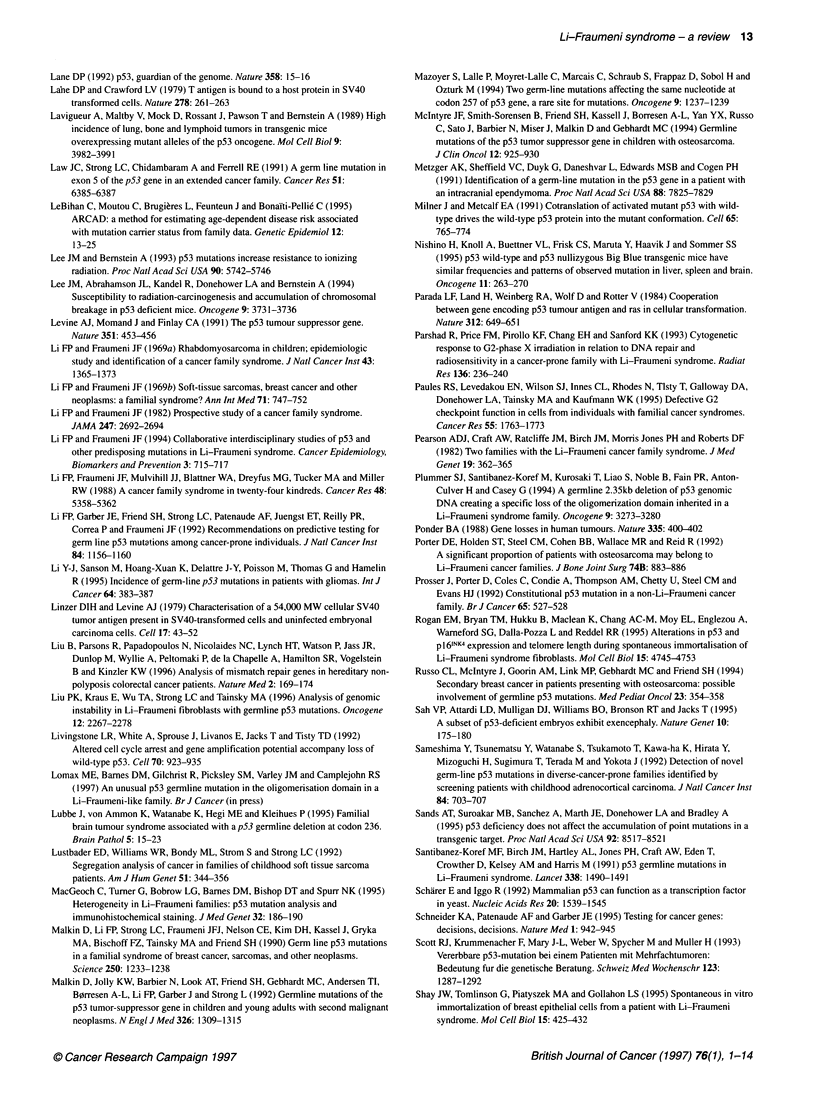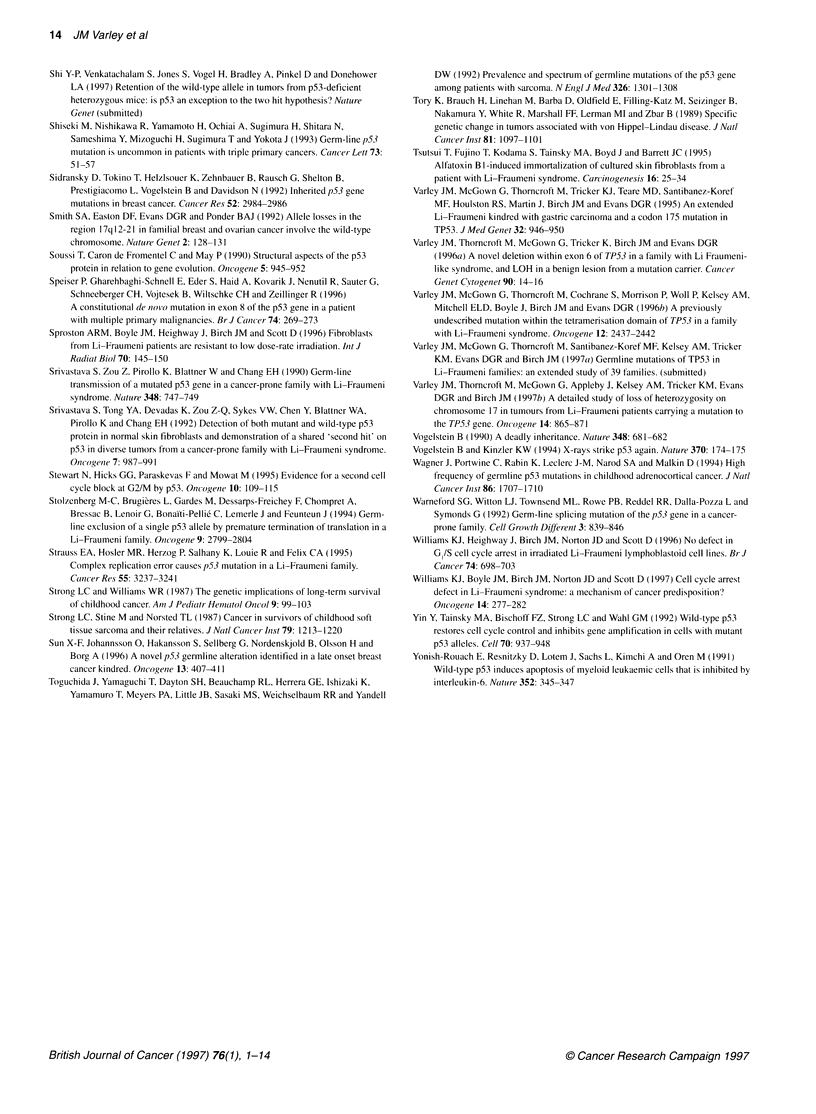# Li-Fraumeni syndrome--a molecular and clinical review.

**DOI:** 10.1038/bjc.1997.328

**Published:** 1997

**Authors:** J. M. Varley, D. G. Evans, J. M. Birch

**Affiliations:** CRC Department of Cancer Genetics, Paterson Institute for Cancer Research, Manchester, UK.


British Journal of Cancer (1997) 76(1), 1–14
© 1997 Cancer Research Campaign

Review

### Li–Fraumeni syndrome – a molecular and clinical review

**JM Varley[1], DGR Evans[1] and JM Birch[2]**

[1]CRC Department of Cancer Genetics, Paterson Institute for Cancer Research, Wilmslow Road, Manchester M20 9BX, UK; [2]CRC Paediatric and Familial Cancer Research Group, Royal Manchester Children's Hospital, Pendlebury, Manchester M27 4HA, UK

In 1969 Li and Fraumeni identified, from a systematic study of 648 children with rhabdomyosarcomas, five families in which a sibling or a cousin was affected by sarcoma (Li and Fraumeni, 1969*a*, *b*). A prospective study of four of the original families indicated that there was a significantly increased risk of cancer within these families, particularly premenopausal breast cancer, as well as a significant excess of second malignancies (Li and Fraumeni, 1982). The term 'Li–Fraumeni syndrome' was used initially by Pearson et al (1982) and is now the most common term for this dominant inherited cancer syndrome. Studies from our own group on a population-based series of children with soft-tissue sarcoma (Birch et al, 1984; 1990) and a USA hospital-based series of survivors of childhood soft-tissue sarcomas from Strong's group in the USA (Strong et al, 1987) confirmed that the familial clustering was due to inherited predisposition and not to environmental factors. Twenty-four families, defined using strict criteria, were subsequently studied by the Li and Fraumeni groups (Li et al, 1988). Such families are now regarded as having 'classic' Li–Fraumeni syndrome (LFS), namely a proband aged under 45 years with a sarcoma having a first-degree relative aged under 45 years with any cancer and an additional first- or second-degree relative aged under 45 years in the same lineage with any cancer or a sarcoma at any age (Li et al, 1988). This study by Li et al (1988) indicated that the spectrum of tumours within the syndrome included acute leukaemia, premenopausal breast carcinoma, brain and adrenocortical tumours as well as bone and soft-tissue sarcomas. Other studies have indicated that a number of other cancers may occur at an increased frequency in these families, notably melanoma, germ cell tumours, Wilms' tumours, gastric and pancreatic carcinomas and lung cancer (Hartley et al, 1987; 1989; Strong et al, 1987; Varley et al, 1995; 1997*a*).

While the definition of classic LFS has become generally accepted, a number of groups have relaxed the description to include incomplete Li–Fraumeni syndrome (a proband with an affected first-degree relative; Brugières et al, 1993) and Li–Fraumeni-like syndrome (LFL). Two groups have defined LFL families independently but using different criteria. Eeles (1995) defined LFL as a clustering of two different tumours (both characteristic of LFS) in individuals who are first- or second-degree relatives of any age. Our own group has defined LFL by more stringent criteria: a proband with any childhood tumour or sarcoma, brain tumour or adrenocortical tumour under 45 years plus a first- or second-degree relative with a typical LFS tumour at any age and another first- or second-degree relative with any cancer under the age of 60 (Birch et al, 1994*a*). In the following discussions, we refer to LFL families using the definition of Birch et al (1994*a*).

The rarity of classic LFS families plus the high mortality within those families precluded a linkage study to identify the likely chromosomal location of the causative genetic defect. Malkin et al (1990) therefore adopted a candidate gene approach and analysed the tumour-suppressor gene *TP53*. This gene was chosen because mutations within *TP53* had been identified in many tumour types, including those commonly associated with Li–Fraumeni syndrome. In addition, studies from transgenic mouse models had indicated that germline mutation to *TP53* was associated with an elevated incidence of sarcomas, lymphoid malignancies, lung and adrenal tumours (Lavigueur et al, 1989). In their original report, Malkin et al (1990) studied five LFS families, all of whose members had mutations within the *TP53* gene.

### THE *TP53* GENE

*TP53* was first identified in 1979 as a gene encoding a protein that complexes to the large T antigen of SV40 (Lane and Crawford, 1979; Linzer and Levine, 1979). A number of properties initially assigned to the TP53 protein, including the ability to immortalize cells (Jenkins et al, 1984) and to transform primary rat embryo fibroblasts in cooperation with *ras* (Eliyahu et al, 1984; Parada et al, 1984), indicated that it was a dominantly acting oncogene. Mutation to *TP53* was found to enhance its transformation efficiency, a finding compatible with the hypothesis that *TP53* is a cellular oncogene that can be activated by mutation (Jenkins et al, 1985). However, a number of observations indicated that the 'wild-type' TP53 proteins that had the properties of oncogenes were in fact mutant, and more detailed studies showed that the wild-type protein could not cooperate with *ras*, and indeed could actually suppress transformation by mutant *TP53* and *ras* (Finlay et al, 1989). Furthermore, *TP53* was shown to be lost or inactivated in a number of malignancies, both human and murine, and is now known to be the most frequently altered gene in human tumours (Hollstein et al, 1991; Levine et al, 1991). There are currently over 4500 reports of somatic mutation in *TP53* in human tumours (Hollstein et al, 1996) and, although the mutations spread over essentially the entire gene, there is considerable clustering of mutations within the central region of the protein. The majority of mutations are missense, with alterations at only five codons representing 25% of all known mutations (codons 175, 245, 248, 249 and 273; Hollstein et al, 1996).

TP53 is now known to possess a spectrum of properties, including transcriptional activation and repression via sequence-specific DNA binding to specific target sequences. Sequences at

*Received 2 December 1996*
*Revised 16 January 1997*
*Accepted 17 January 1997*

*Correspondence to:* JM Varley

**Table 1** Details of germline *TP53* mutations reported in the literature

| Codon | Exon | Mutation | Type[a] | Type of family[b] | Reference | Loss of heterozygosity (LOH) |
|---|---|---|---|---|---|---|
| 220 | 6 | TAT→TGT Tyr→Cys | T | LFS | Birch et al (1994*a*) | LOH in 1/2 |
| 175 | 5 | CGC→CAC Arg→His | CpG | LFS | Birch et al (1994*a*) | No LOH in 3/4, LOH of mutant allele 1/4 |
| 248 | 7 | CGG→CAG Arg→Gln | CpG | LFS | Birch et al (1994*a*) | LOH in 1/4 |
| 248 | 7 | CGG→CAG Arg→Gln | CpG | LFS | Birch et al (1994*a*) | LOH in 1/3 |
| 248 | 7 | CGG→TGG Arg→Trp | CpG | LFS | Birch et al (1994*a*) | LOH in 1/3 |
| 108–111 | 4 | Complex deletion-insertion | Del/ins | LFS | Birch et al (1994*a*) | |
| 180 | 5 | GAG→AAG Glu→Lys | T | LFL | Birch et al (1994*a*) | LOH of mutant allele 1/1 |
| 136 | 5 | CAA→TAA Gln→Stop | T | LFS | Varley et al (1997*a*) | LOH in 1/2 |
| 191 | 6 | 2-bp Deletion prem stop | Del | LFL | Varley et al (1996) | LOH in 5/6 |
| 248 | 7 | CGG→CAG Arg→Gln | CpG | LFS | Varley et al (1997*a*) | No LOH in 3/3 |
| 344 | 10 | CTG→CCG Leu→Pro | T | LFS | Varley et al (1996) | LOH in 1/1 |
| 273 | 8 | CGT→CAT Arg→His | CpG | LFL | Varley et al (1997*a*) | No LOH in 1/1 |
| 245 | 7 | GGC→AGC Gly→Ser | CpG | LFL | Varley et al (1997*a*) | |
| 273 | 8 | CGT→TGT Arg→Cys | CpG | LFS | Varley et al (1997*a*) | LOH in 1/1 |
| 175 | 5 | CGC→CAC Arg→His | CpG | LFS | Varley et al (1997*a*) | LOH in 3/4 |
| Exon 1 | 1 | 167-bp Deletion | Del | LFS | Varley et al (1997*a*) | LOH in 1/1 |
| Intron 3 | | Splice acceptor ag/T→aa/T | T | LFS | Varley et al (1997*a*) | |
| Exon 4 | 4 | Splice donor CG/g→CA/g | CpG | LFS | Varley et al (1997*a*) | |
| 209 | 6 | AGA→TGA Arg→Stop | V | LFS | Varley et al (1997*a*) | |
| 248 | 7 | CGG→CAG Arg→Gln | CpG | LFS | Frebourg et al (1995) | |
| 213 | 6 | CGA→TGA Arg→Stop | CpG | LFS | Frebourg et al (1995) | |
| 275 | 8 | TGT→TAT Cys→Tyr | T | LFS | Frebourg et al (1995) | |
| 248 | 7 | CGG→CAG Arg→Gln | CpG | LFS | Frebourg et al (1995) | |
| Intron 4 | | Splice donor gt→at | T | LFS | Frebourg et al (1995) | |
| 273 | 8 | CGT→TGT Arg→Cys | CpG | LFS | Frebourg et al (1995) | |
| 175 | 5 | CGC→CAC Arg→His | CpG | LFS | Frebourg et al (1995) | |
| 193 | 6 | CAT→CGT His→Arg | T | LFS | Frebourg et al (1995) | |
| 215 | 6 | 1-bp deletion leading to stop | Del | LFS | Stolzenberg et al (1994) | |
| Exon 10 | 10 | Complex deletion of | Del | LFS | Plummer et al (1994) | LOH in 1/1 |
| 248 | 7 | CGG→TGG Arg→Trp | CpG | LFS | Malkin et al (1990) | |
| 258 | 7 | GAA→AAA Glu→Lys | T | LFS | Malkin et al (1990) | LOH in 1/1 |
| 245 | 7 | GGC→TGC Gly→Cys | V | LFS | Malkin et al (1990) | LOH in 1/1 |
| 248 | 7 | CGG→TGG Arg→Trp | CpG | LFL | Malkin et al (1990) | |
| 252 | 7 | CTC→CCC Leu→Pro | T | LFS | Malkin et al (1990) | |
| 248 | 7 | CGG→CAG Arg→Gln | CpG | NFH | Toguchida et al (1992) | |
| 241 | 7 | TCC→TTC Ser→Phe | T | NFH (new mutation) | Toguchida et al (1992) | |
| 245 | 7 | GGC→AGC Gly→Ser | CpG | FH | Toguchida et al (1992) | |
| 151–152 | 5 | 1-bp Insertion stop at 180 | Ins | LFS | Toguchida et al (1992) | |
| 209–210 | 6 | 2-bp Deletion stop at 214 | Del | LFS | Toguchida et al (1992) | |
| 71–72 | 4 | 1-bp Insertion stop at 148 | Ins | FH | Toguchida et al (1992) | |
| 120 | 4 | AAG→TAG Lys→Stop | V | FH | Toguchida et al (1992) | |
| 282 | 8 | CGG→TGG Arg→Trp | CpG | LFL | Toguchida et al (1992) | |
| 245 | 7 | GGC→AGC Gly→Ser | CpG | LFL | MacGeoch et al (1995) | |
| 273 | 8 | CGT→TGT Arg→Cys | CpG | LFL | Eeles et al (1993) | LOH 1/3, LOH of mutant allele in 1/3 |
| 267 | 8 | CGG→CAG Arg→Gln | CpG | FH | Prosser et al (1992) | No LOH at p53, LOH at YNZ22 |
| 245 | 7 | GGC→GAC Gly→Asp | T | LFS | Srivastava et al (1990) | LOH in 4/4 |
| 242 | 7 | CGT→CAT Cys→Tyr | CpG | LFL | Metzger et al (1991) | No LOH in 1/1 |
| 248 | 7 | CGG→TGG Arg→Trp | CpG | FH | Malkin et al (1992) | |
| 282 | 8 | CGG→TGG Arg→Trp | CpG | FH | Malkin et al (1992) | |
| 273 | 8 | CGT→CAT Arg→His | CpG | NFH | Malkin et al (1992) | |
| 325 | 9 | GGA→GTA Gly→Val | V | NFH | Malkin et al (1992) | |
| Intron 5 | | Splicing mutation 11bp del | Del | NFH | Felix et al (1993) | LOH in 1/1 |
| 273 | 8 | CGT→CAT Arg→His | CpG | LFS? | Kovar et al (1992) | LOH in 1/1 |
| 248 | 7 | CGG→TGG Arg→Trp | CpG | FH | Brugieres et al (1993) | |
| 273 | 8 | CGT→GGT Arg→Gly | V | LFS | Brugieres et al (1993) | |
| 282 | 8 | CGG→TGG Arg→Trp | CpG | FH | Brugieres et al (1993) | |
| 133 | 5 | ATG→ACG Met→Thr | T | LFS | Law et al (1991) | |
| 215 | 6 | 1-bp Deletion prem stop 246 | Del | LFS | Hamelin et al (1994) | LOH in 1/1 |
| 257 | 7 | CTG→CAG Leu→Gln | V | FH | Mazoyer et al (1994) | |
| 257 | 7 | 1-bp Deletion prem stop | Del | LFL | Mazoyer et al (1994) | |
| Exon 4 | 4 | Splice donor CG/g→CA/g | CpG | LFL | Warneford et al (1992) | No LOH in 1/1 |
| 196 | 6 | CGA→TGA Arg→Stop | CpG | FH | Grayson et al (1994) | LOH in 2/2 |
| 152 | 5 | CCG→CTG Pro→Leu | CpG | FH | Wagner et al (1994) | |
| 219 | 6 | CCC→TCC Pro→Ser | T | NFH | Wagner et al (1994) | |
| 235 | 7 | AAC→GAC Asn→Asp | T | NFH | Wagner et al (1994) | |
| 175 | 5 | CGC→CAC Arg→His | CpG | NFH | Kyritsis et al (1994) | |
| 305 | 8 | AAG→ATG Lys→Met | V | NFH | Kyritsis et al (1994) | |
| 175 | 5 | CGC→CAC Arg→His | CpG | NFH | Kyritsis et al (1994) | |
| 272 | 8 | GTG→GCG Val→Ala | T | Same patient as above | Kyritsis et al (1994) | |

 *© Cancer Research Campaign 1997*

**Table 1** cont.

| Codon | Exon | Mutation | Type[a] | Type of family[b] | Reference | Loss of heterozygosity (LOH) |
|---|---|---|---|---|---|---|
| 181 | 5 | CGC→CTC Arg→Leu | V | NFH | Kyritsis et al (1994) | |
| 138 | 5 | GCC→TCC Ala→Ser | V′ | LFL | Kyritsis et al (1994) | |
| 155 | 5 | ACC→AAC Thr→Asn | V | FH | Kyritsis et al (1994) | |
| 167 | 5 | CAG→AAG Gln→Lys | V · | NFH | Kyritsis et al (1994) | |
| 174 | 5 | AGG→GGG Arg→Gly | T | NFH | Kyritsis et al (1994) | |
| 241 | 7 | TCC→ACC Ser→Thr | V | NFH | Kyritsis et al (1994) | |
| 213 | 6 | CGA→TGA Arg→Stop | CpG | FH | Horio et al (1994) | LOH in 1/3 |
| 175 | 5 | CGC→CAC Arg→His | CpG | NFH | McIntyre et al (1994) | |
| 175 | 5 | CGC→CAC Arg→His | CpG | LFS | McIntyre et al (1994) | |
| 163 | 5 | TAC→TGC Tyr→Cys | T | FH | McIntyre et al (1994) | |
| 209 | 6 | AGA→TGA Arg→Stop | V | NFH | McIntyre et al (1994) | |
| 242 | 7 | TGC→TAC Cys→Tyr | T | NFH | McIntyre et al (1994) | |
| 273 | 8 | CGT→AGT Arg→Ser | V | NFH | McIntyre et al (1994) | |
| 271 | 8 | GAG→GTG Glu→Val | V | NFH | McIntyre et al (1994) | |
| 154 | 5 | GGC→GTC Gly→Val | V | NFH? | Chen et al (1995) | LOH in 1/1 |
| 256 | 7 | ACA→ATA Thr→Ile | T | NFH? | Chen et al (1995) | Mutation at codon 280 in other allele in tumour |
| 151 | 5 | CCC→TCC Pro→Ser | T | NFH (new mutation) | Gutierrez et al (1994) | LOH in 1/1 |
| 181 | 5 | CGC→TGC Arg→Cys | CpG | FH | Sidransky et al (1992) | |
| 181 | 5 | CGC→CAC Arg→His | CpG | FH | Børresen et al (1992) | LOH in 1/1 |
| 245 | 7 | GGC→AGC Gly→Ser | CpG | FH | Børresen et al (1992) | |
| Intron 5 | | Splice donor ag/G→cg/G | V | LFL | Jolly et al (1994) | LOH in 1/1 |
| 242 | 7 | TGC→TAC Cys→Tyr | T | NFH | Russo et al (1994) | |
| 272 | 8 | GTG→TTG Val→Leu | V | LFS | Felix et al (1992) | No LOH in 1/1 |
| 293 | 8 | GGG→TGG Gly→Trp | V | NFH | Chung et al (1991) | LOH in 1/1 |
| 282 | 8 | CGG→TGG Arg→Trp | CpG | NFH | Iavarone et al (1992) | LOH in 1/1 |
| 282 | 8 | CGG→TGG Arg→Trp | CpG | LFS | Shiseki et al (1993) | |
| 248 | 7 | CGG→TGG Arg→Trp | CpG | LFS | Scott et al (1993) | No LOH in 1/1 |
| 235 | 7 | AAC→AGC Asn→Ser | T | NFH | Diller et al (1995) | |
| 306 | 8 | CGA→CCA Arg→Pro | V | NFH | Diller et al (1995) | |
| 227 | 7 | TCT→ACT Ser→Thr | V | NFH | Diller et al (1995) | |
| 307 | 8 | 1-bp Deletion prem stop | Del | LFL/S | Sameshima et al (1992) | LOH in 1/1 |
| 286 | 8 | GAA→GCA Glu→Ala | V | LFL | Sameshima et al (1992) | No LOH in 2/2 |
| 273 | 8 | CGT→CAT Arg→His | CpG | LFS | Porter et al (1992) | |
| 245 | 7 | GGC→AGC Gly→Ser | CpG | FH? | Felix et al (1995) | LOH in 1/1 |
| 236 | 7 | Deletion of codon (del TAC) | Del | LFL | Lubbe et al (1995) | LOH in 3/4 |
| 215–218 | 6 | AGT GTG→AGT TGG TTG Val→Trp Leu | Del/ins | LFS | Strauss et al (1995) | |
| 248 | 7 | CGG→TGG Arg→Trp | CpG | FH | Bang et al (1995) | |
| 251 | 7 | ATC→ATG Ile→Met | V | FH | Li et al (1995) | |
| 233 | 7 | CAC→GAC His→Asn | V | NFH | Li et al (1995) | |
| 273 | 8 | CGT→TGT Arg→Cys | CpG | FH | Li et al (1995) | |
| 245 | 7 | GGC→AGC Gly→Ser | CpG | NFH | Li et al (1995) | LOH in 1/1 |
| 133 | 5 | ATG→ACG Met→Thr | T | LFS | Shay et al (1995) | |
| 82 | 4 | CCG→CTG Pro→Leu | CpG | FH | Sun et al (1996) | (No LOH, but not sure of significance of mutation) |
| 278 | 8 | CCT→CTT Pro→Leu | T | NFH | Speiser et al (1996) | LOH in 5/7 |
| 209 | 6 | 2-bp Deletion | Del | NFH | Felix et al (1996) | |

[a]Mutation type: CpG, transition at CpG; T, non-CpG transition; V, transversion; del/ins, deletion and/or insertion. [b]Family history: LFS and LFL, classic Li–Fraumeni syndrome and Li–Fraumeni-like respectively; see text for definition. FH, family history but not conforming to LFS or LFL, taken as the patient having at least one first-degree relative with cancer at age under 60 years; NFH, no strong family history.

the amino-terminus of the protein have transactivation properties when interacting with target sequences through specific DNA interactions (Fields and Jang, 1990). Five highly conserved domains have been identified through sequence comparison across species (Soussi et al, 1990), and these have been termed domains I–V. Conserved domain I is within the transactivation domain. The central part of the protein (amino acids 100–293) encodes the DNA-binding domain, which recognizes and binds a consensus target sequence (El-Deiry et al, 1992). Conserved domains II–V all reside within this core DNA-binding region. The crystal structure of the core DNA-binding domain has been determined as a large β-sandwich acting as a scaffold for two large loops and a loop–sheet–helix, which together make all the interactions with DNA (Cho et al, 1994). Of tremendous interest from this study was the observation that the loop domains overlap with the highly conserved domains and that the majority of mutations in human tumours fall within the three loop motifs. Three residues (K120, C277 and R280) interact through hydrogen bonds with DNA bases and five (K120, S241, R273, A276 and R283) interact with phosphate groups of the major groove. The arginine residue at codon 248 has four crucial interactions with phosphate groups along the minor groove (Cho et al, 1994; Vogelstein and Kinzler, 1994; Arrowsmith and Morin, 1996). Two classes of mutation are predicted from knowledge of this structure (Cho et al, 1994).

© Cancer Research Campaign 1997

Contact mutations affect residues that directly contact DNA, including R248 and R273, which are among the most frequently mutated residues. Structural mutations affect residues that do not directly contact DNA but which stabilize the structure of TP53, for example R249 and R175, both of which connect two of the loops.

The C-terminal end of the core DNA-binding domain was shown by Cho et al (1994) to extend from the major groove away from the bulk of the DNA-binding domain. TP53 functions as a tetramer, and residues 324–355 are responsible for oligomerization. The structure of this domain has been deduced from nuclear magnetic resonance (Clore et al, 1994) and X-ray crystallography (Jeffrey et al, 1995) and reveals that the TP53 tetramer is actually a dimer of dimers. This structure fits well the observation that the TP53 binding consensus sequence is a pair of inverted repeats (El-Deiry et al, 1992). Relatively few mutations have been seen in the tetramerization domain, the majority of which lead to premature termination of the protein (Hollstein et al, 1996).

The *TP53* gene maps to chromosome 17p13.1, spanning around 20 kilobase pairs (kb), and comprises 11 exons. The coding region spans exons 2–11, with the core DNA-binding domain being encoded by part of exon 4 through to exon 8. As most of the somatic mutations have been found within this core domain, many groups have only analysed exons 5–8 for point mutation. While there is little doubt that mutations within these exons are the most frequent, it has become difficult to assess the frequency of mutations outside this region, and it has been estimated that over 20% mutations remain undetected (Greenblatt et al, 1994).

In addition to the properties of TP53 described above, wild-type TP53 has been termed the 'guardian of the genome' (Lane, 1992). Wild-type TP53 functions in checkpoint control after DNA damage (Kuerbitz et al, 1992), resulting in either a delay in cell cycle progression at the $G_1$/S border to allow DNA repair (Kastan et al, 1991) or apoptosis (Yonish-Rouach et al, 1991). TP53 has also been implicated directly in DNA repair (Ford and Hanawalt, 1995) and in $G_2$ arrest (Paules et al, 1995; Stewart et al, 1995).

The majority of mutations described in other tumour-suppressor genes such as *RB1*, *APC* and *BRCA1* are clearly loss-of-function mutations. The mutation spectrum in these genes shows a high proportion of nonsense and frameshift mutations that produce an absent or truncated protein. Mutations to *TP53* are most frequently missense and, while they may lead to loss of certain functions such as failure to arrest in $G_1$/S or $G_2$, they frequently result in the gain of a function such as the ability to cooperate with *ras* in the transformation of fibroblasts. In addition, certain TP53 mutants may demonstrate a dominant-negative function by, for example, forming oligomeric complexes with wild-type TP53 and blocking its normal functions (Milner and Metcalf, 1991).

The relatively bewildering spectrum of mutations seen in human tumours coupled to some understanding of the functions of various domains of the TP53 protein has led a number of groups to establish assays to evaluate the relevance of mutations. Many assays are available, including growth arrest of target cells, apoptosis or transactivation of target genes in mammalian cells. Perhaps the most useful assays, and the ones which have additionally been used as a screen to detect the presence of *TP53* mutations in both human tumours and the germline, are functional assays in yeast. Initially the assay, based on the inability of the majority of TP53 mutants to transactivate target genes, was established in human cells (Frebourg et al, 1992). However with the demonstration that mammalian TP53 can function in yeast cells (Schärer and Iggo, 1992), a simple functional assay was described (Ishioka et al, 1993; Flaman et al, 1995). This assay will now provide a mechanism by which mutations can be assessed for their transactivation properties. As yet, relatively few mutations have been assessed in this yeast assay, but not all mutations will be detected. The assay relies on high-quality mRNA from the test source, which is reverse-transcribed into cDNA and integrated into a yeast plasmid by homologous recombination in vivo. Codons 67–347 are integrated into the yeast plasmid, therefore any mutations outside these regions will not be detected. In addition, large deletions or insertions may not be detected and nor will any mutations in which there is little or no expression of the mutant transcript (Flaman et al, 1995).

### *TP53* MUTATIONS AND LI–FRAUMENI FAMILIES

In the initial study describing the involvement of germline *TP53* mutations in LFS, Malkin et al (1990) studied five Li–Fraumeni families and sequenced exons 5–8 in affected individuals from each family. All five families had missense mutations, all within the highly conserved region IV of exon 7. The clustering of mutations within this short region of the protein was confirmed in a further family (Srivastava et al, 1990), leading to the proposal that there was a restriction upon the mutations in the *TP53* gene that are permitted in the germline (Vogelstein, 1990). However, analysis of eight classic LFS families by Santibanez-Koref et al (1991) revealed that mutations in exon 7 only occurred in two families, and subsequently mutations have been found throughout the gene (see below). Although *TP53* mutations have been reported in over 50 Li–Fraumeni families (see Table 1), only two studies have analysed consecutively ascertained series of such families. Frebourg et al (1995) have studied 15 families conforming to classic LFS and have identified eight families with germline mutations by screening exons 2–11. Recent data from our own studies (Varley et al, 1997*a*), in which all 11 exons plus the promoter region and all splice junctions have been analysed by direct sequencing, has identified 15 germline *TP53* mutations in 21 LFS families (71%) and four mutations in 18 LFL families. From these two studies, it is clear that germline mutations within the coding regions of *TP53* are not responsible for all Li–Fraumeni families.

### GERMLINE *TP53* MUTATIONS IN PATIENTS WITH TUMOURS TYPICAL OF LFS

Since the original description of germline *TP53* mutations, there have been a number of studies in which series of patients with tumours characteristic of Li–Fraumeni syndrome have been screened to determine the proportion of those patients who are gene carriers (see Table 1). Patients with bone or soft-tissue sarcomas were studied by Toguchida et al (1992), including 181 cases of sporadic disease and 15 patients with an unusual family history of cancer or multiple primary tumours. Three *TP53* germline mutations were detected in the former group, but all were subsequently found to have multiple primary tumours or first-degree relatives with sarcomas. In the group that had been selected, five germline mutations were found (33%), indicating that up to one-third of the patients with sarcoma and either multiple tumours or an unusual family history were carriers of germline *TP53* mutations. McIntyre et al (1994) studied 237 unselected children with osteosarcoma and found seven with a germline mutation; Porter et al (1992) reported one germline mutations out of seventeen patients with osteosarcoma and Iavarone et al (1992) reported one germline mutation in four patients with multifocal osteogenic sarcoma. Diller et al

 © Cancer Research Campaign 1997

(1995) examined 33 patients with childhood rhabdomyosarcoma and found three germline mutations, all in the group of 13 children under the age of three (23%). Clearly it is now possible to identify a group of affected individuals with sarcoma at high risk of carrying a germline *TP53* mutation. The occurrence of sarcomas (particularly rhabdomyosarcomas) in young children, an unusual family history or multiple primary tumours should warrant an examination for a germline *TP53* mutation. From studies already published, it appears that up to one-third of this group may be carriers.

A number of studies have also been carried out on patients with brain tumours. Kyritsis et al (1994) found *TP53* mutations in nine of 51 patients with glioma, all of whom had either multifocal disease or a family history of cancer. Other studies have found germline *TP53* mutations in around 2–10% of cases of brain tumour, with the highest frequency in gliomas in children or young adults with an unusual personal or family history of cancer (Chung et al, 1991; Chen et al, 1995; Felix et al, 1995; Li et al, 1995).

Premenopausal breast cancer is very common in Li–Fraumeni families, and three groups have examined series of sporadic breast cancer patients for germline *TP53* mutations (Børresen et al, 1992; Prosser et al, 1992; Sidransky et al, 1992). Only four mutations were detected in a total of 499 patients studied by these three groups, indicating that *TP53* mutations are responsible for no more than 1% of breast tumours. All four patients showed very strong family histories of cancer. Although frequently overlooked, patients with germline *TP53* mutations contribute significantly to the number of breast cancer cases with a strong inherited component, particularly when breast cancer is diagnosed at a very young age (< 30 years). It has been estimated that around 1% of breast cancer cases between the ages of 30 and 40 years arise within Li–Fraumeni families (Easton et al, 1993). This is clearly lower than the proportion of early-onset cancers due to either *BRCA1* or *BRCA2* but is nonetheless significant.

Wagner et al (1994) examined children with adrenocortical carcinoma (ACC) and, although the numbers are small, three of six had germline *TP53* mutations. Selection of families on the basis of an ACC also indicated a very high frequency of mutations (Sameshima et al, 1992). In the Li–Fraumeni families that we have studied to date, all five in which there is an adrenocortical tumour have a germline *TP53* mutation, and there are no cases of ACC in any negative family (Varley et al, 1997*a*).

Finally, studies of patients selected on the basis of multiple primary tumours (Malkin et al, 1992; Shiseki et al, 1993; Russo et al, 1994) have shown that they have germline *TP53* mutations at a frequency of between 7% and 20%.

Although Li–Fraumeni families are rare, nonetheless germline *TP53* mutations are frequent in individuals with specific cancers, particularly at young age. Half of all cases of childhood ACC and between one-quarter and one-third of childhood sarcomas, particularly multifocal or with a family history, arise in patients with a germline *TP53* mutation. Any patient presenting with such tumours, especially if there is additional personal or family history of malignancy, should be considered as a potential mutation carrier. This could have profound implications for both the treatment of the patients and their subsequent monitoring and for analysing *TP53* for mutation with a view to offering other family members predictive tests. This will be discussed in more detail later in this review. It should also be noted that in the majority of studies cited above, analysis of *TP53* has not covered the entire coding region and, if this were carried out, the frequency of germline mutations within various groups of cancer patients may rise.

### THE SPECTRUM AND TYPE OF GERMLINE *TP53* MUTATIONS

There are now over 50 families reported that conform to the definition of classic LFS or LFL, using the definition of Birch et al (1994*a*), and in whose members germline *TP53* mutations have been found (Table 1). From the literature, the proportion of families that do not have a detectable coding mutation is not generally clear. There have been only two studies in which there has been a systematic analysis of *TP53* in series of families (Birch et al, 1994*a*; Frebourg et al, 1995; Varley et al, 1997*a*), and both of these studies have identified families in which there is no coding mutation. Frebourg et al (1995) found 7 of 15 LFS families to have no mutation, although they did not examine exon 1 or the promoter region. In our own series (Varley et al, 1997*a*), we found mutations in 71% of classic LFS families. There are many reports in the literature of single families in which a mutation has been found, but it is not possible to determine how many families have been examined and found to be negative; however, it seems likely that at least 30% of LFS families have no coding mutation. There is only one definite report of a Li–Fraumeni family in which the syndrome was unlinked to *TP53* (Birch et al, 1994*b*), although normal tissues from affected individuals within this family have been shown to express elevated levels of *TP53*. The only other family in which there appeared to be no *TP53* mutation but overexpression in normal tissues (Barnes et al, 1992) has been subsequently shown to have a mutation in the tetramerization domain encoded by exon 10 (Lomax et al, 1997). There have been reports that in some negative families the expression of *TP53* is limited to one allele, implicating a possible regulatory mutation in some Li–Fraumeni families (Li and Fraumeni, 1994).

We are aware of 114 reports of germline *TP53* mutations (see Table 1), ascertained by analysis of LFS or LFL families, as well as others such as breast (Børresen et al, 1992; Prosser et al, 1992; Jolly et al, 1994; Shay et al, 1995; Sun et al, 1996) or brain (Lubbe et al, 1995) site-specific families or cohorts of patients with tumours typical of Li–Fraumeni syndrome (see above). The spectrum of mutations shows that the majority of all germline mutations cluster in exons 5–8, however this is undoubtedly skewed because most studies only examine these exons. In our own study, of 19 mutations found to date, five fall outside these exons (26%; Varley et al, 1997*a*). This finding has profound implications for genetic testing within Li–Fraumeni families, and we recommend that all exons, both coding and non-coding, plus all splice junctions and the promoter region are analysed.

We have analysed all the reported germline mutations to determine the mutation type. There is no difference in the frequency of missense and nonsense mutations between Li–Fraumeni families, other types of families and individuals with no family history. In addition, there are no differences between LFS and LFL families.

There are two reports of large germline deletions, one involving the whole of exon 10 (Plummer et al, 1994) and the other removing 167 bp, including part of exon 1 and intron 1 (Varley et al, 1997*a*). Both of these deletions were flanked by short direct repeat sequences. Thirteen other mutations involve short insertion or deletion events (Sameshima et al, 1992; Toguchida et al, 1992; Felix et al, 1993; Birch et al, 1994*a*; Hamelin et al, 1994; Mazoyer et al, 1994; Stolzenberg et al, 1994; Lubbe et al, 1995; Strauss et al, 1995; Felix et al, 1996; Varley et al, 1996*a*, 1997*a*). All of these events occurred where there were runs of two or more identical bases or short repeated motifs, except in one case (Mazoyer et al,

© Cancer Research Campaign 1997

1994). Although deletions and insertions are most frequent in exons 2–4 and 9–11 in sporadic tumours (Harris, 1996), in the 114 germline mutations we have reviewed, the situation is not as straightforward. There is only one report of a mutation in exon 1 (Varley et al, 1997*a*), which is a large deletion, and there are no reported mutations in exons 2 and 3. Two of the six mutations in exon 4 are deletions or insertions. One of the three mutations reported in exons 9–11 is a large deletion (Plummer et al, 1994). Deletions or insertions therefore constitute a significant proportion of the mutations in exons 1–4 and 9–11 in germline tumours, as seen in sporadic tumours. However, six of the 14 mutations reported in exon 6 are deletions or insertions at short repeat sequences, with all except one leading to a frameshift and premature termination. Harris (1996) suggests that deletions or insertions in sporadic tumours are more frequent in exons 2–4 and 9–11 because the regions of the TP53 protein encoded by these exons generally require more than one mutation to abrogate function, and this is most efficiently caused by a deletion or insertion event. Why these events are seen at such a high frequency in exon 6 as germline mutations is unclear at present. Thirteen of the 15 deletion or insertion events were found in patients with a strong family history, twelve with family histories compatible with Li–Fraumeni syndrome (see Table 1).

In all reports of germline *TP53* mutations, transitions are the most common change, accounting for 66% of all germline mutations, with those within CpGs being the predominant type (44% of all mutations). These types of mutation are considered to represent endogenous events. Cytosine and 5-methylcytosine can spontaneously deaminate to uracil and thymine, respectively, resulting in G:C to A:T transitions. The majority of transitions occur at CpG dinucleotides, which are common sites for methylation, and transitions can also occur as a result of replication errors. When the frequency of transitions in patients with any family history of cancer is compared with that in patients with no family history, there is an under-representation of transitions at CpG in the latter group ($P < 0.004$). In this and subsequent analyses, we scored patients as having a family history if they had at least one first-degree relative with cancer under the age of 60 years or if they had a family history compatible with LFS or LFL. There was no difference in the frequency of transitions between patients with a family history compatible with LFS or LFL and all other patients with or without a family history.

Transversions represent 21% of all germline mutations. Transversions can occur as a result of replication errors but are also considered to represent the possibility of exogenous carcinogenic insult. G residues are a common target for bulky carcinogen-induced adducts, resulting in stalling of the replication machinery and misincorporation by DNA polymerase. We therefore analysed the data on the 114 germline mutations to determine whether there was a difference between the frequency of transversions in patients with a strong family history and those without a family history. Whereas only 11 transversions were seen in 81 cases (14%) when there was a strong family history, 13 of the other 33 cases (without a family history) had transversion mutations (39%). This difference is statistically significant ($P < 0.005$) and raises the possibility that the majority of germline mutations that have arisen recently may be due to exposure to carcinogens. A more detailed collaborative epidemiological study would be required to address this possibility. Intriguingly, when patients with germline mutations are separated into LFS or LFL family members vs others (both with and without a family history), the difference in the frequency of transversions is still significantly different ($P < 0.02$), and an increased occurrence of deletions or insertions in LFS or LFL families reaches significance ($P < 0.03$).

### GENOTYPE–PHENOTYPE CORRELATIONS AND PENETRANCE

The finding that around 30% of classic LFS families and a higher percentage of LFL families have no *TP53* coding mutation raises the question as to whether there may be phenotypic differences between those families with and those without identifiable mutations. Although there are numerous reports of individual families or patients, or groups of families and patients, with germline *TP53* mutations, there are only two published series of families in whom the entire coding sequence of *TP53* has been analysed (Frebourg et al, 1995; Varley et al, 1997*a*), with only the latter study including an analysis of exon 1 and the promoter region. Furthermore, in the study by Frebourg et al (1995), details of the pedigrees of the families included in the study were not published. The only series in which it is possible to analyse the cancer phenotype in LFS and LFL families with and without germline *TP53* mutations is that assembled by our own group. In 30 of the families in whom sequence analysis of the entire *TP53* gene has been completed (18 LFS and 12 LFL), histopathological review of tumours has been carried out. The distribution of cancers by morphological type and age at diagnosis has been analysed in these families. The analysis included cancers in the probands and their first- and second-degree relatives. Twelve of the 18 LFS and four of the 12 LFL families carried germline *TP53* mutations. In these 16 families, mutation status was determined in as many family members as possible, but all cancers in eligible relatives as defined above were included in the analysis, regardless of whether or not mutation status had been determined in the individual concerned. This strategy ensured comparability between the mutation-positive and -negative families. In total, 116 cancers in LFS and LFL families have been included in the analysis (Table 2).

When the distribution of cancer cases in the 30 LFS and LFL families overall is analysed, there was a higher proportion of sarcomas in LFS families, but this can be attributed to the requirement of the classic LFS criteria for a proband with sarcoma. If sarcomas are excluded, then the distribution of first primary

**Table 2** Spectrum and distribution of cancers in LFS and LFL families with and without germline *TP53* mutations

| Tumour type | *TP53* positive | *TP53* negative | Total |
|---|---|---|---|
| Soft-tissue sarcoma[a] | 13 (19.1) | 10 (20.8) | 23 (19.8) |
| Bone[a] | 7 (10.3) | 5 (10.4) | 12 (10.3) |
| Breast[b] | 19 (27.9) | 13 (27.1) | 32 (27.6) |
| Brain | 13 (19.1) | 3 (6.3) | 16 (13.8) |
| Leukaemia/lymphoma | 1 (1.5) | 5 (10.4) | 6 (5.2) |
| Adrenocortical | 5 (7.4) | 0 | 5 (4.3) |
| Digestive system | 7 (10.3) | 6 (12.5) | 13 (11.2) |
| Genitourinary system | 1 (1.5) | 4 (8.3) | 5 (4.3) |
| Lung | 2 (2.9) | 2 (4.2) | 4 (3.5) |
| Total | 68 (100) | 48 (100) | 116 (100) |

[a]Eighty-five per cent of sarcomas (soft tissue plus bone) in *TP53*-positive families occurred under the age of 20 years compared with 50% of those in *TP53*-negative families. [b]Seventy-nine per cent of breast cancers in *TP53*-positive families occurred under the age of 40 years compared with 39% in *TP53*-negative families. Numbers in parentheses are percentages.

© Cancer Research Campaign 1997

tumours in LFS and LFL families is very similar. In LFS families, 40% of the non-sarcoma cancers were carcinomas of the breast compared with 39% in LFL families; the figures for brain tumours were 18% and 22% respectively. In both sets of families, the age distribution at diagnosis of cancer was strikingly young. In LFS families, 56% of the cancer cases were diagnosed under age 30 years and 100% under age 50 years; in LFL families the figures were 44% and 78% respectively. In the general population, only 2% of cancer cases occur under 30 years of age and 11% under 50 years of age (data derived from cancer registrations for England and Wales, 1991, Office of National Statistics, September 1996).

When the two sets of families were divided on the basis of the presence or absence of a germline *TP53* mutation, it was found that overall the cancer cases in families with germline *TP53* mutations had a somewhat earlier age of onset, but this was not particularly marked (median age of onset of cancer in LFS and LFL families with germline *TP53* mutations is 27 years and in those without mutations is 31 years). The proportions of sarcomas and breast cancers in the *TP53*-positive and -negative families are very similar (sarcomas in positive vs negative families is 29% vs 31%, respectively, and breast cancers 28% vs 27% respectively; see Table 2). However, the age distributions of these cancer cases in the two sets of families did differ. Thus 85% of soft tissue sarcomas in mutation-positive families were diagnosed when the individual was under 20 years of age compared with 50% in the mutation-negative families. For breast cancer, 79% were diagnosed under 40 years in the mutation-positive families compared with 39% in the negative families.

Considering other types of cancers, there was a distinct excess of brain tumours in the *TP53*-positive families, 19% of all cancers in the mutation-positive families were brain tumours compared with only 6% in the negative families, and 81% of all brain tumours in the entire series of families occur in those with germline *TP53* mutations. All five adrenocortical tumours in the series occurred in families with mutations. However, there appeared to be a deficit of leukaemias and lymphomas in the mutation-positive families, with less than 2% of the cancers in these families being of these types compared with 10% in the mutation-negative families.

The above analyses were based on first primary cancers only. The occurrence of multiple primary cancers in members of Li–Fraumeni families is well described, and there was a similar proportion of individuals with second and subsequent primary tumours in the *TP53* mutation-positive and -negative families. However, in the mutation-positive families sarcoma was the most common second primary, whereas in the negative families breast cancer was the most common.

The data reported above are incomplete as diagnostic review of cancers in the entire series of families is still in progress. However, on the basis of these preliminary analyses there do appear to be phenotypic differences between Li–Fraumeni families with and without germline *TP53* mutations. 'Marker' cancers for the presence of such mutations in both LFS and LFL families appear to be early-onset soft-tissue sarcoma, in particular rhabdomyosarcoma diagnosed under the median age of diagnosis for all childhood rhabdomyosarcomas (under 4 years), adrenocortical carcinoma, breast cancer aged under 40 years, and brain tumours in children and young adults. Histological examination of the CNS tumours that occurred in carriers of *TP53* germline mutations in our series showed them to be mainly high-grade astrocytoma or glioblastoma and childhood medulloblastoma. Results of the formal analysis of cancer phenotype in the entire series of families examined by us may have important implications for counselling and future screening regimens in families both with and without germline *TP53* mutations.

In those families with germline *TP53* mutations, it will also be important in relation to genetic counselling and disease screening to have reliable estimates of penetrance and age-specific risks for the various component cancers of the syndrome. At present, there are insufficient data to provide such estimates. However, preliminary data do exist that could provide some guidance. On the basis of population studies of cancer risks in families of children with sarcoma and follow-up studies in families with LFS, it has been shown that the risk of cancer is elevated up to, but not beyond, age 60 years (Birch et al, 1990; Garber et al, 1991). Using a data set comprising details of occurrence of cancer among the close relatives of a hospital-based series of children who had survived sarcoma, Lustbader et al (1992) conducted a segregation analysis. This demonstrated that the distribution of cancer in the families was consistent with a rare autosomal dominant gene (frequency 0.00002) with penetrance of approximately 50% by age 40 years and up to 90% by age 60 years. The age-specific penetrance was higher in women than in men because of breast cancer occurrence. It is likely that the major component of the familial cancer clustering detected by Lustbader et al (1992) was due to *TP53* but there is a possibility that other genes such as *BRCA2* and *NF1* may be making small contributions. This should be borne in mind when interpreting these results.

The only published estimates of age-specific risks associated with germline *TP53* mutations were based on only five families but used a new method which addresses the problem of ascertainment bias (LeBihan et al, 1995). Risks were estimated to be 42% between ages 0 and 16 years, 38% between ages 17 and 45 years and 63% after age 45 years; lifetime risk was estimated to be 85% (LeBihan et al, 1995). Although these results should be viewed with caution, because they are based on such small numbers, the method should prove to be useful in analysing larger series. This requires international collaboration, and such a study is planned.

### LOSS OF HETEROZYGOSITY STUDIES IN TUMOURS FROM PATIENTS WITH GERMLINE *TP53* MUTATIONS

Wild-type *TP53* is considered to be a tumour-suppressor gene (Hollstein et al, 1991; Levine et al, 1991; Lane, 1992) and as such a number of tumours of a variety of types from patients with germline *TP53* mutations have been analysed for loss of heterozygosity (LOH). LOH at a particular locus/gene is considered to indicate the presence of a tumour suppressor at that locus (Ponder, 1988), with the LOH event unmasking the recessive mutation. Tumour-suppressor genes such as *BRCA1* (Smith et al, 1992), *RB1* (Cavenee et al, 1983), *BRCA2* (Gudmundsson et al, 1995), *APC* (Ichii et al, 1992) and *VHL* (Tory et al, 1989) have been studied in many families in which there is a germline mutation to those genes, and loss of the wild-type allele has been seen at high frequency in the tumours of patients carrying the mutation. All these genes therefore fulfil the criteria expected of tumour-suppressor genes, namely obeying Knudson's 'two-hit' hypothesis (Knudson, 1971), whereby one defective allele is inherited and the second is inactivated by loss of part or all of the wild-type chromosome (Ponder, 1988).

To our knowledge 82 tumours from patients with germline mutations at *TP53* have been studied for LOH, with LOH detected

© Cancer Research Campaign 1997

in 57% of those tumours (see Table 2; Varley et al, 1997*b*). This figure is lower than expected from a comparison of the loss of other tumour-suppressor genes in inherited cancer syndromes, in which levels of 80% LOH are not unusual. However, only one systematic study of LOH has been carried out in a large series of patients with defined *TP53* mutations in tumour material that has been characterized according to current standard criteria (Varley et al, 1997*b*); most other studies have involved examination of LOH in a single family and/or a very few tumours. In the former study, LOH was observed in only 44% of tumours, but a number of interesting patterns of LOH were described. All tumours except breast in which there was a codon 248 mutation showed retention of the wild-type allele. Only one other tumour described in the literature with a codon 248 mutation has been analysed for LOH and that also failed to show LOH (Scott et al, 1993). This finding is discussed in more detail in Varley et al (1997*b*), but it is not obvious why there should be no loss of the wild-type allele in the presence of a codon 248 mutation, whereas a variety of tumours with a range of other mutations do show LOH. If tumours with codon 248 mutations are excluded from the analysis, LOH is seen in 60%, which is still lower than that seen for other tumour-suppressor genes. However, it is possible that there is under-reporting of LOH, with contamination of the tumour sample with normal cells accounting for the number of cases in which there is apparently no LOH. In the consecutive series in which all tumours were assessed to ensure a high percentage of tumour cells before analysis, LOH was also seen in only 60% of cases (excluding those with a codon 248 mutation; Varley et al, 1997*b*). The figure of 60% LOH is therefore consistent among the different studies and reflects the true level of LOH.

LOH has been seen in a number of benign or premalignant lesions, including ductal carcinoma in situ of the breast and endometrial hyperplasia (Varley et al, 1996*a*) and an adreno-cortical tumour (Varley et al, 1995). More surprisingly, loss of the mutant allele has been seen in three cases (Eeles et al, 1993; Varley et al, 1997*b*).

To date, of four reports in which LOH has been examined in gastric/stomach tumours, all showed retention of the wild-type allele (Scott et al, 1993; Horio et al, 1994; Varley et al, 1997*b*). In contrast, of thirteen breast tumours studied, LOH was seen in ten (Malkin et al, 1990; Børresen et al, 1992; Prosser et al, 1992; Srivastava et al, 1992; Warneford et al, 1992; Eeles et al, 1993; Varley et al, 1997*b*). We have examined other markers on chromosome 17 in five breast tumours showing LOH at *TP53* and have demonstrated that the loss seen is not targeting more distal candidate tumour-suppressor loci on the short arm of chromosome 17 nor loci on the long arm (Varley et al, 1997*b*).

The relatively low level of LOH seen in tumours from patients with germline *TP53* mutations compared with the levels of loss seen of other genes in inherited cancer syndromes is not entirely surprising given the range of functions of TP53 as described above. It could be considered that gain-of-function mutants or those showing dominant-negative features may be sufficient to induce tumour formation in the presence of the wild-type gene. Why this should be so in tumours in patients with an inherited mutation when the situation is different in sporadic tumours remains unclear. Recent data from studies of *TP53*-deficient mice demonstrate that 40–50% of tumours that develop in heterozygous null mice do not show LOH and that the retained allele has the properties of wild-type *TP53* (LA Donehower, personal communication; Shi et al, 1997).

### FUNCTIONAL STUDIES OF LI-FRAUMENI CELLS

There have been many studies carried out to examine the altered function(s) of the variety of *TP53* mutations that occur in human tumours. Many of these studies involve the introduction of mutant *TP53* constructs into cells that either contain an endogenous wild-type *TP53* gene or that are null for *TP53*. Frequently the mutant *TP53* gene is overexpressed, and a number of functional end points are examined. These studies all suffer from a common drawback: the genetic background in which the assays take place does not reflect that of a normal cell from a patient with a germline *TP53* mutation. For this reason, a number of groups have examined cells derived from patients with germline *TP53* mutations, most commonly lymphoblastoid or fibroblast cell lines.

Fibroblasts from Li–Fraumeni patients show chromosomal sensitivity (measured by an increased frequency of chromosome aberrations) to the effects of ionizing radiation (Parshad et al, 1993), in contrast to the cellular radioresistance (measured by cell survival) observed by Bech-Hansen et al (1981) and our own group (Sproston et al, 1996). Chromosomal radiosensitivity has also been observed in lymphocytes from Li–Fraumeni patients (D Scott, personal communication). While Li–Fraumeni fibroblasts show a level of transient $G_1$ arrest that is indistinguishable from normal cells, their permanent $G_1$ arrest is considerably reduced (Williams et al, 1997). Permanent $G_1$ arrest in fibroblasts is considered to be the functional equivalent of apoptosis in other cell types, in removing cells that have received genetic damage. This hypothesis is supported by the findings of Camplejohn et al (1995) that lymphocytes from Li–Fraumeni patients with a germline *TP53* mutation show an abnormal apoptotic response. The failure of Li–Fraumeni fibroblasts to permanently arrest at $G_1$ may contribute towards the predisposition of heterozygous individuals to cancer.

The above studies were all carried out on relatively early-passage heterozygous (i.e. wild type/mutant) fibroblasts that had been treated with a DNA-damaging agent of some sort, usually irradiation. However Li–Fraumeni fibroblasts that are maintained in culture and not subjected to any damaging agents also show a range of abnormalities. As they are cultured through progressive population doublings, they show an increased number of chromosome abnormalities of all types (Bischoff et al, 1990; Yin et al, 1992; Rogan et al, 1995), loss of the wild-type allele (Yin et al, 1992; Rogan et al, 1995), changes in morphology and, rarely, spontaneous immortalization (Bischoff et al, 1990; Rogan et al, 1995) accompanied by telomere elongation (Rogan et al, 1995). Spontaneous immortalization has also been reported in breast epithelial cells from a LFS patient with a germline *TP53* mutation (Shay et al, 1995), and even Li–Fraumeni cells that fail to immortalize spontaneously can be induced to do so by treatment with various agents that have no effect on normal cells (Shay et al, 1995; Tsutsui et al, 1995).

The loss of wild-type *TP53* from Li–Fraumeni fibroblasts correlates in vitro with failure to arrest in $G_1$ when treated with a uridine biosynthesis inhibitor PALA and subsequent PALA-selected gene amplification (Livingstone et al, 1992; Yin et al, 1992). This phenotype is reversible upon reintroducing a wild-type *TP53* construct into the fibroblasts. PALA reduces the intracellular pool of nucleotides, and inappropriate entry of cells into S-phase after PALA treatment as a result of the failure to arrest in $G_1$ could result in chromosome breakage or fragmentation and could initiate gene amplification (Yin et al, 1992), which is one manifestation of genomic instability. PK Liu et al (1996) have examined other

 © Cancer Research Campaign 1997

aspects of genomic instability in LFS fibroblast cells that are early wild type/mutant or late passage (–/mutant). Interestingly in fibroblasts that still retain the wild-type allele, there is a high level of point mutation within a reporter plasmid, and in fact this level is higher than in those cells that have lost the wild-type allele. The authors suggest that heterozygous LFS cells contain an activity (a mutant *TP53* gene) that promotes mutations, contributing towards the genomic instability seen in these cells (PK Liu et al, 1996).

The demonstration of a failed $G_2$ arrest in Li–Fraumeni fibroblasts (Paules et al, 1995) is further evidence of a failure to recognize DNA damage. Even partial failure of a $G_2$ checkpoint in Li–Fraumeni cells would be sufficient to allow some damaged cells to enter mitosis. As these cells could well have chromosome damage, populations of chromosomally abnormal cells could expand. The data obtained from the studies of human cells are supported by observations on *TP53*-deficient mice. These mice have defective $G_2$ (Bouffler et al, 1995) and mitotic spindle checkpoint control (Cross et al, 1995), leading to numerical chromosome changes (both aneuploidy and polyploidy). In summary, Li–Fraumeni cells with a mutant *TP53* gene have lost control at a number of levels of genome surveillance. Not only has normal TP53 function been lost in terms of transactivation, interaction with other cellular proteins and DNA binding, but both key cell cycle checkpoints are perturbed. The failure of Li–Fraumeni cells carrying a germline *TP53* defect to recognize DNA damage and to arrest at either checkpoint, coupled with a potential 'mutator' phenotype (PK Liu et al, 1996) will result in cells with a steadily increasing burden of genetic damage. Whether the extent of these phenotypic alterations is different in a variety of cell types remains to be investigated, but it could account for the tumour spectrum seen in Li–Fraumeni families.

Studies using EBV-transformed lymphoblastoid cell lines derived from patients with germline *TP53* mutations have shown that these cells behave in a manner indistinguishable from normal cells. Lalle et al (1995) showed that the cells retained a normal induction of $p21^{waf1}$ and MDM2 upon DNA damage and maintained genomic stability and that there was no loss of the wild-type *TP53* allele in heterozygous cells even after 1 year in culture. Williams et al (1996) showed that the presence of a heterozygous *TP53* mutation had no effect on TP53 expression after DNA damage or on $G_1$/S arrest. There are clearly differences in the phenotype of unstimulated lymphocytes from patients with germline *TP53* mutations, such as an abnormal apoptotic response (Camplejohn et al, 1995), and it is possible that the immortalization by EBV is influencing the behaviour of these cells in a TP53-independent manner. However, the data may also suggest that the different tissue-specific phenotypic responses to the presence of a heterozygous *TP53* mutation could account for the spectrum of tumour types observed in patients from Li–Fraumeni families (Lalle et al, 1995; Williams et al, 1996).

To summarize all the data obtained from many different studies, the presence of a germline *TP53* mutation in the heterozygous state appears to permit the accumulation of genetic damage, leading to genomic instability and eventually neoplastic transformation. However, although a range of tumour types is seen in Li–Fraumeni families, many common solid tumours are not represented to a significant degree (e.g. colorectal tumours and gynaecological malignancies), and the reason for the limited spectrum of tumours remains unclear.

One important feature of all the above studies in Li–Fraumeni cells is that they are extremely sensitive to DNA damage of any sort. This has considerable implications for the clinical management of patients in Li–Fraumeni families, particularly in screening for cancers and for treatment. While the frequency of radiation-induced tumours is still not known in germline mutant *TP53* carriers, there is evidence that second malignant neoplasms occur at a relatively high frequency in the radiation zone (Strong and Williams, 1987; Heyn et al, 1993), although more extensive studies must be carried out to confirm this. The relative contributions of genetic background and radiation remain to be evaluated in a careful epidemiological study.

### ANIMAL MODELS OF LI–FRAUMENI SYNDROME

The earliest study in which a *TP53*-transgenic mouse was generated was described in 1989 (Lavigueur et al, 1989), before the genetic basis of LFS was elucidated. The observed occurrence of multiple tumours in the transgenic strains, including tumours typical of LFS, contributed towards the decision to study *TP53* as a candidate gene by Malkin et al (1990). These transgenic mice carried their normal wild-type *TP53* alleles as well as high numbers of copies of a mutant transgene, and tumours developed in 20% of the transgenic mice, commonly osteosarcomas and lung adenocarcinomas but also lymphomas and a range of other tumours. Interestingly, the levels of expression of the mutant transgenes did not correlate simply with the sites of tumour formation.

Donehower et al (1992) reported the generation of transgenic knockout mouse strains that were homozygous null for *TP53*, with part of intron 4 and exon 5 replaced with a *neo* gene. These mice were, surprisingly, viable and initially appeared to have no developmental abnormalities. Three-quarters of the homozygous null mice developed tumours by 6 months of age, predominantly lymphoblastic lymphomas but also sarcomas. The same group (Harvey et al, 1993) extended these studies to examine heterozygous null/wild-type mice, which reflects more accurately the situation in some LFS families. In contrast to the situation in homozygous null mice in which 100% had developed tumours or died by 10 months of age, only 50% of heterozygotes had developed tumours by the age of 18 months. The spectrum of tumours differed between the two groups, with 58% of the heterozygotes developing sarcomas (predominantly osteosarcomas) and only 32% developing lymphomas (Harvey et al, 1993), but this animal model is still not a perfect LFS paradigm, as brain and mammary tumours are very rarely seen. The heterozygotes showed considerably increased susceptibility to dimethylnitrosamine carcinogenesis compared with wild-type mice. Loss of heterozygosity was seen in only 55% of tumours, although the retained allele had not been sequenced in those tumours showing no LOH. This frequency is close to that seen in tumours of patients with germline *TP53* mutations (see above and Varley et al, 1997*b*). Using a different disrupted *TP53* gene in which 40% of the gene had been replaced, Jacks et al (1994) reported essentially identical results to Donehower's group. Subsequently two groups reported that homozygous null mice do not develop normally, with up to 30% of female embryos showing abnormal neural tube closure and exencephaly (Armstrong et al, 1995; Sah et al, 1995).

*TP53*-deficient mice are extremely sensitive to gamma irradiation with a reduced tumour latency following low-level irradiation (Kemp et al, 1994). This sensitivity is associated with an increase in radiation-induced double-strand chromosomal breaks (Lee et al, 1994). Transgenic mice carrying mutant *TP53* genes show cellular radioresistance in haematopoietic cells (Lee and Bernstein, 1993).

© Cancer Research Campaign 1997

At least two studies have used a *lacI* transgene as a target to determine the mutation rate in wild-type and homozygous null mice. Neither study has been able to demonstrate an increased mutation frequency, either spontaneously or after treatment with DNA-damaging agents (Nishino et al, 1995; Sands et al, 1995).

In many respects the transgenic and *TP53*-deficient mice are good models for Li–Fraumeni syndrome. However none of the mouse strains generated to date is a perfect model, particularly with respect to the spectrum of tumours seen in these animals. The targeted introduction of a specific point mutation into one allele in a heterozygous mouse would be of great interest.

### CLINICAL ASPECTS OF LI–FRAUMENI FAMILIES

The definition of classic LFS, like the Amsterdam criteria for HNPCC (B Liu et al, 1996), seems to be a good predictor for mutations in the causative genes *TP53* and the HNPCC mismatch repair genes respectively. The definition is therefore useful in genetic counselling as it allows the counsellor to talk in purely Mendelian terms. In our series (Varley et al, 1997*a*), *TP53* mutations have been detected in 71% of families with classic LFS, and it is possible that a high proportion of families with no detectable *TP53* mutation have functional loss. Our data, together with those of other groups (Sameshima et al, 1992; Wagner et al, 1994; Diller et al, 1995), suggest that the chance of finding a germline *TP53* mutation in a family with LFS increases if the family includes a case of ACC and/or rhabdomyosarcoma diagnosed in early childhood. This allows the clinician to talk with reasonable certainty about the possibility of predictive tests in the family. Even in families excluded from *TP53* by linkage, another dominant gene is likely; therefore it is reasonable to talk of 50% risk of transmission of a highly penetrant cancer-predisposing gene with a high risk of childhood and early adult malignancy. In LFS families, the risk of childhood tumours is estimated to be in the order of 20%, with 50% developing cancer by 40 years and 90% by 60 years of age (Lustbader et al, 1992).

LFL families are more difficult. Using our own definition (Birch et al, 1994*a*), under 25% will have a detectable *TP53* mutation. A proportion may be due to other genes, but some may be due to chance association. It is not possible, therefore, to counsel in terms of a 50% risk for relatives of affected cases or to make firm predictions about the likely tumour spectrum for at-risk individuals. Some of these family aggregates may be due to a recent new mutation and some latitude with the criteria, particularly if the family includes childhood ACC or rhabdomyosarcoma, may well predict a high proportion of the *TP53*-positive LFL families. Once a mutation has been detected genetic counselling becomes straightforward, but the absence of a mutation does not allow complete reassurance.

In common with current practice in breast cancer families, predictive presymptomatic testing should not be offered to at-risk individuals unless a *TP53* mutation has already been identified in an affected individual. Although mutations can be found in DNA extracted from paraffin-embedded tumour material, the DNA is often of poor quality. The DNA may fail to amplify certain exons of *TP53* and, if non-sequencing mutation detection methods are used, certain mutations can be missed (see Varley et al, 1995). Tumours may also of course contain a somatic mutation and this possibility would have to be excluded by finding the identical mutation in multiple tumours from the same or related individuals. Only if the mutation was confirmed in this way could it be used in predictive testing. In practice, therefore, it is usually necessary to have a living affected individual on whom to undertake mutation analysis. As described above, 26% of mutations occur outside the accepted hotspots, and therefore the whole gene needs to be sequenced in each case. Mutations that truncate the protein or that are accepted as affecting key properties of TP53 from functional studies, or their presence either in other Li–Fraumeni families or commonly in human tumours, can be used in predictive testing. However predictive testing using missense or splice-site mutations outside the above categories should be undertaken with extreme caution. Even if other affected family members test positive for the same sequence variant, this could still represent a rare polymorphism and, if possible, some functional studies should be carried out to determine relevance of the alteration. However, if many affected family members are positive for the 'mutation' and, especially if there is loss of the wild-type allele in tumour tissue, then testing would be reasonable.

Our own early experience and that of others (Schneider et al, 1995) would suggest that uptake of testing will be relatively low. Once an individual is availed of the risks to a mutation carrier and the paucity of options with regard to early detection and prevention, they may prefer to live with the better-than-even chance that they will not carry the mutation. (Their 50% risk will have been reduced, assuming they have reached adulthood by the time of counselling.) Nonetheless a significant minority proceed to testing and will require extra support both psychologically and in terms of extra surveillance if they test positive. It is vital that individuals are given adequate time for reflection before taking the test and therefore testing protocols should be similar to those adopted for Huntington's disease (Li et al, 1992; Eeles, 1993; Birch, 1994). It is widely accepted that testing of children should not be offered routinely; however, parents may exert considerably pressure to have a child tested. Each case should be treated on its own merits. If the case can be answered that the child is likely to benefit from the test, then after very careful counselling that includes the child or children, proceeding to testing may be appropriate in some isolated cases. We have been involved in tests on four children over 10 years of age, one of whom was mutation positive. Although follow-up is still early, all the children and families seem to have benefited from testing. Nonetheless, long-term effects such as insurance and other discrimination on *TP53*-positive individuals may deter most genetic counsellors and parents from testing children.

Given the wide diversity and unpredictability of tumours in Li–Fraumeni syndrome, no widely accepted screening procedure has been devised. It may be wise to avoid as far as possible any radiological screening that exposes the patient to excessive ionizing radiation. However one of the few organs that could be targeted for screening, i.e. the breast (Li et al, 1992), does not have another proven screening test. Ultrasound is very operator dependent and has poor sensitivity, while magnetic resonance imaging (MRI) is only now being evaluated. Mammography would have to be undertaken annually from 20 years if it were to be used and, while very little radiation passes beyond the skin, concern must be voiced about the possibility of inducing a tumour that would otherwise not have occurred.

While there is no proven way of detecting tumours early in Li–Fraumeni syndrome, it is vital that individuals at risk have access to informed clinicians and that early symptoms are rigorously investigated. We offer an annual clinical review to all at-risk individuals and, in addition, we offer annual abdominal ultrasound

 *© Cancer Research Campaign 1997*

and full blood count to children and a specialist breast examination with or without ultrasound to women over 20 years. Selective screening, such as cranial MRI in families with brain tumours or gastroscopy in families with gastric carcinoma, could also be justified, although their effectiveness is not known.

Some women in LFS families have undertaken preventative mastectomy (DGRE, unpublished data). However this does not allow the same level of reassurance as in *BRCA1* and *BRCA2* carriers as so many other organs are at risk. Families are therefore pinning a great deal of hope on genetic therapies. Germline gene therapy, even if it were possible, has not been approved by any regulatory authority. Even selective/tissue-specific targeting of treatment is still beyond our current capabilities. Nonetheless, the outcome of many clinical trials involving the manipulation of *TP53* is eagerly awaited. This will probably have more implications for treatment of tumours than in prevention, but any procedure reducing mortality in Li–Fraumeni syndrome would be gratefully accepted by the families. There is a general assumption that tumours in carriers of germline *TP53* mutations may be relatively radiation resistant, although hard data are not available and there appears to be a high risk of second malignancy in the radiation field (Strong and Williams, 1987; Heyn et al, 1993). However, more extensive longitudinal studies on germline *TP53* mutation carriers are necessary to assess the effectiveness of current treatment regimens.

It should be stressed that counselling, genetic testing and screening for early detection of cancers in Li–Fraumeni families are in their infancy. Although some data are available on age-specific risks of cancers associated with Li–Fraumeni syndrome, at present the estimated risks are based on very small numbers of cancer-affected individuals. Furthermore, families in whom *TP53* mutation analysis has been undertaken were selected because of their striking patterns of cancer. In the circumstances, it is recommended that any such counselling, testing and screening should only be undertaken in centres experienced with both clinical aspects of the syndrome and with expertise in sequence analysis of the entire *TP53* gene. Of equal importance, families in whom *TP53* mutation analysis is undertaken should be included in a research programme encompassing molecular genetic, genetic epidemiological, clinical and psychosocial aspects. It is only by applying such a systematic approach and with international collaboration that progress will be made.

### CONCLUDING COMMENTS

Although families conforming to the strict definitions of classic LFS or LFL are very rare, germline *TP53* mutations may be relatively common in certain groups of cancer patients. The definition of classic LFS is still of great value in clinical practice and predictive testing but may need to be modified to take into account the occurrence of tumours such as adrenocortical carcinomas. In view of the potentially damaging effects of radiation on cells with *TP53* mutations, considerable care should be taken when screening patients with germline mutations for tumours, and the design and implementation of novel screening protocols is of some urgency. Studies of cells from patients with germline mutations have provided, and will continue to provide, insight into the function(s) of TP53, and further characterization of families with no detectable coding mutation may identify germline mutations in other genes involved in the same pathway(s).

### ACKNOWLEDGEMENTS

We would like to thank our many colleagues who have contributed to the work described from our own laboratories. We would also like to thank Professor David Harnden and Drs David Scott, Karen Tricker and Geoff Margison for helpful comments on the manuscript.

### REFERENCES

Armstrong JF, Kaufman MH, Harrison DJ and Clarke AR (1995) High-frequency developmental abnormalities in p53-deficient mice. *Curr Biol* **5**: 937–943

Arrowsmith CH and Morin P (1996) New insights into p53 function from structural studies. *Oncogene* **12**: 1379–1385

Bang YJ, Kang SH, Kim TY, Jung CW, Oh SM, Choe KJ and Kim NK (1995) The first documentation of Li–Fraumeni syndrome in Korea. *J Korean Med Sci* **10**: 205–210

Barnes DM, Hanby AM, Gillett CE, Mohammed S, Hodgson S, Bobrow LG, Leigh IM, Purkis T, MacGeoch C, Spurr NK, Bartek J, Vojtesek B, Picksley SM and Lane DP (1992) Abnormal expression of wild-type p53 protein in normal cells of a cancer family patient. *Lancet* **340**: 259–263

Bech-Hansen NT, Blattner WA, Sell BM, McKee EA, Lampkin BC, Fraumeni JF and Paterson MC (1981) Transmission of *in vitro* radioresistance in a cancer-prone family. *Lancet* **1**: 1335–1337

Birch JM (1994) Li–Fraumeni syndrome. *Eur J Cancer* **30A**: 1935–1941

Birch JM, Hartley AL, Marsden HB, Harris M and Swindell R (1984) Excess risk of breast cancer in the mothers of children with soft tissue sarcomas. *Br J Cancer* **49**: 325–331

Birch JM, Hartley AL, Blair V, Kelsey AH, Harris M, Teare MD and Morris Jones PH (1990) Cancer in the families of children with soft tissue sarcomas. *Cancer* **66**: 2237–2248

Birch JM, Hartley AL, Tricker KJ, Prosser J, Condie A, Kelsey AM, Harris M, Morris Jones PH, Binchy A, Crowther D, Craft AW, Eden OB, Evans DGR, Thompson E, Mann JR, Martin J, Mitchell ELD and Santibanez-Koref MF (1994*a*) Prevalence and diversity of constitutional mutations in the *p53* gene among 21 Li–Fraumeni families. *Cancer Res* **54**: 1298–1304

Birch JM, Heighway J, Teare MD, Kelsey AM, Hartley AL, Tricker KJ, Crowther D, Lane DP and Santibanez-Koref MF (1994*b*) Linkage studies in a Li–Fraumeni family with increased expression of p53 protein but no germline mutation in p53. *Br J Cancer* **70**: 1176–1181

Bischoff FZ, Yim SO, Pathak S, Grant G, Siciliano MJ, Giovanella BC, Strong LC and Tainsky MA (1990) Spontaneous abnormalities in normal fibroblasts from patients with Li–Fraumeni cancer syndrome: aneuploidy and immortalization. *Cancer Res* **50**: 7979–7984

Børresen A-L, Andersen TI, Garber J, Barbier-Piraux N, Thorlacius S, Eyfjörd J, Ottestad L, Smith-Sorensen B, Hovig E, Malkin D and Friend SH (1992) Screening for germ line *TP53* mutations in breast cancer patients. *Cancer Res* **52**: 3234–3236

Bouffler SD, Kemp CJ, Balmain A and Cox R (1995) Spontaneous and ionizing radiation-induced chromosomal abnormalities in *p53*-deficient mice. *Cancer Res* **55**: 3883–3889

Brugières L, Gardes M, Moutou C, Chompret A, Meresse V, Martin A, Poisson N, Flamant F, Bonaïti-Pellié C, Lemerle J and Feunteun J (1993) Screening for germ line p53 mutations in children with malignant tumours and a family history of cancer. *Cancer Res* **53**: 452–455

Camplejohn RS, Perry P, Hodgson SV, Turner G, Williams A, Upton C, MacGeoch C, Mohammed S and Barnes DM (1995) A possible screening test for inherited p53-related defects based on the apoptotic response of peripheral blood lymphocytes to DNA damage. *Br J Cancer* **72**: 654–662

Cavenee WK, Dryja TP, Phillips RA, Benedict WF, Godbout R, Gallie BL, Murphree AL, Strong LC and White RL (1983) Expression of recessive alleles by chromosomal mechanisms in retinoblastoma. *Nature* **305**: 779–784

Chen P, Iavarone A, Fick J, Edwards M, Prados M and Israel MA (1995) Constitutional p53 mutations associated with brain tumors in young adults. *Cancer Genet Cytogenet* **82**: 106–115

Cho Y, Gorina S, Jeffrey PD and Pavletich NP (1994) Crystal structure of a p53 tumor suppressor-DNA complex: understanding tumorigenic mutations. *Science* **265**: 346–355

Chung R, Whaley J, Kley N, Anderson K, Louis D, Menon A, Hettlich C, Freiman R, Hedley-White ET, Martuza R, Jenkins R, Yandell D and Seizinger BR (1991) *TP53* gene mutations and 17p deletions in human astrocytomas. *Genes Chrom Cancer* **3**: 323–331

© Cancer Research Campaign 1997

Clore GM, Omichinski JG, Sakaguchi K, Zambrano N, Sakamoto H, Appella E and Gronenborn AM (1994) High-resolution structure of the oligomerization domain of p53 by multidimensional NMR. *Science* **265**: 386–391

Cross SM, Sanchez CA, Morgan CA, Schimke MK, Ramel S, Idzerda RL, Raskind WH and Reid BJ (1995) A p53-dependent mouse spindle checkpoint. *Science* **267**: 1353–1356

Diller L, Sexsmith E, Gottlieb A, Li FP and Malkin D (1995) Germline p53 mutations are frequently detected in young children with rhabdomyosarcoma. *J Clin Invest* **95**: 1606–1611

Donehower LA, Harvey M, Slagle BL, McArthur MJ, Montgomery CA, Butel JS and Bradley A (1992) Mice deficient for p53 are developmentally normal but susceptible to spontaneous tumours. *Nature* **356**: 215–221

Easton D, Ford D and Peto J (1993) Inherited susceptibility to breast cancer. *Cancer Surv* **18**: 95–113

Eeles RA (1993) Predictive testing for germline mutations in the p53 gene: are all the questions answered? *Eur J Cancer* **29A**: 1361–1365

Eeles RA (1995) Germline mutations in the *TP53* gene. *Cancer Surv* **25**: 101–123

Eeles RA, Warren W, Knee G, Bartek J, Averill D, Stratton MR, Blake PR, Tait DM, Lane DP, Easton DF, Yarnold JR, Cooper CS and Sloane JP (1993) Constitutional mutation in exon 8 of the p53 gene in a patient with multiple primary tumours: molecular and immunohistochemical findings. *Oncogene* **8**: 1269–1276

El-Deiry WS, Kern SE, Pietenpol JA, Kinzler KW and Vogelstein B (1992) Definition of a consensus binding site for p53. *Nature Genet* **1**: 45–49

Eliyahu D, Raz A, Gruss P, Givol D and Oren M (1984) Participation of p53 cellular tumour antigen in transformation of normal embryonic cells. *Nature* **312**: 646–649

Felix CA, Nau MM, Takahashi T, Mitsudomi T, Chiba I, Poplack DG, Reaman GH, Cole DE, Letterio JJ, Whang-Peng J, Knutsen T and Minna JD (1992) Hereditary and acquired p53 gene mutations in childhood acute lymphoblastic leukemia. *J Clin Invest* **89**: 640–647

Felix CA, Strauss EA, D'Amico D, Tsokos M, Winter S, Mitsudomi T, Nau MM, Brown DL, Leahey AM, Horowitz ME, Poplack DG, Costin D and Minna JD (1993) A novel germline p53 splicing mutation in a pediatric patient with a second malignant neoplasm. *Oncogene* **8**: 1203–1210

Felix CA, Slavc I, Dunn M, Strauss EA, Phillips PC, Rorke LB, Sutton L, Bunin GR and Biegel JA (1995) p53 gene mutations in pediatric brain tumors. *Med Pediat Oncol* **25**: 431–436

Felix CA, Hosler MR, Provisor D, Salhany K, Sexsmith EA, Slater DJ, Cheung N-KV, Winick NJ, Strauss EA, Heyn R, Lange BJ and Malkin D (1996) The p53 gene in pediatric therapy-related leukemia and myelodysplasia. *Blood* **87**: 4376–4381

Fields S and Jang SK (1990) Presence of a potent transcription activating sequence in the p53 protein. *Science* **249**: 1046–1048

Finlay CA, Hinds PW and Levine AJ (1989) The p53 proto-oncogene can act as a suppressor of transformation. *Cell* **57**: 1083–1093

Flaman J-M, Frebourg T, Moreau V, Charbonnier F, Martin C, Chappuis P, Sappino A-P, Limacher J-M, Bron L, Benhattar J, Tada M, Van Meir EG, Estreicher A and Iggo RD (1995) A simple p53 functional assay for screening cell lines, blood, and tumours. *Proc Natl Acad Sci USA* **92**: 3963–3967

Ford JM and Hanawalt PC (1995) Li-Fraumeni syndrome fibroblasts homozygous for p53 mutations are deficient in global DNA repair but exhibit normal transcription-coupled repair and enhanced UV-resistance. *Proc Natl Acad Sci USA* **92**: 8876–8880

Frebourg T, Barbier N, Kassel J, Ng Y-S, Romero P and Friend SH (1992) A functional screen for germ line *p53* mutations based on transcriptional activation. *Cancer Res* **52**: 6976–6978

Frebourg T, Barbier N, Yan Y, Garber JE, Dreyfus M, Fraumeni J, Li FP and Friend SH (1995) Germ-line p53 mutations in 15 families with Li–Fraumeni syndrome. *Am J Hum Genet* **56**: 608–615

Garber JE, Goldstein AM, Kantor AF, Dreyfus MG, Fraumeni JF and Li FP (1991) Follow-up study of twenty-four families with Li–Fraumeni syndrome. *Cancer Res* **51**: 6094–6097

Grayson GH, Moore S, Schneider BG, Saldivar V and Hensel CH (1994) Novel germline mutation of the p53 tumor suppressor gene in a child with incidentally discovered adrenal cortical carcinoma. *Am J Pediatr Hematol Oncol* **16**: 341–347

Greenblatt MS, Bennett WP, Hollstein M and Harris CC (1994) Mutations in the *p53* tumour suppressor gene: clues to cancer etiology and molecular pathogenesis. *Cancer Res* **54**: 4855–4878

Gudmundsson J, Johannesdottir G, Bergthorsson JT, Arason A, Ingvarsson S, Egilsson V and Barkardottir R (1995) Different tumor types from BRCA2 carriers show wild-type chromosome deletions on 13q12–q13. *Cancer Res* **55**: 4830–4832

Gutierrez MI, Bhatia KG, Barreiro C, Spangler G, Schvartzmann E, Muriel FS and Magrath IT (1994) A *de novo* p53 germline mutation affecting codon 151 in a six year old child with multiple tumors. *Hum Mol Genet* **3**: 2247–2248

Hamelin R, Barichard F, Henry I, Junien C and Thomas G (1994) Single base pair germ-line deletion in the p53 gene in a cancer predisposed family. *Hum Genet* **94**: 88–90

Harris CC (1996) The 1995 Walter Hubert lecture – molecular epidemiology of human cancer: insights from the mutational analysis of the p53 tumour-suppressor gene. *Br J Cancer* **73**: 261–269

Hartley AL, Birch JM, Marsden HB and Harris M (1987) Malignant melanoma in families of children with osteosarcoma, chondrosarcoma and adrenal cortical carcinoma. *J Med Genet* **24**: 664–668

Hartley AL, Birch JM, Kelsey AM, Marsden HB, Harris M and Teare MD (1989) Are germ cell tumours part of the Li–Fraumeni cancer family syndrome. *Cancer Genet Cytogenet* **42**: 221–226

Harvey M, McArthur MJ, Montgomery Jr CA, Butel JS, Bradley A and Donehower LA (1993) Spontaneous and carcinogen-induced tumorigenesis in p53-deficient mice. *Nature Genet* **5**: 225–229

Heyn R, Haeberlen V, Newton WA, Ragab AH, Raney B, Tefft M, Wharam M, Ensign LG and Maurer HM (1993) Second malignant neoplasms in children treated for rhabdomyosarcoma. *J Clin Oncol* **11**: 262–270

Hollstein M, Sidransky D, Vogelstein B and Harris CC (1991) p53 mutations in human cancers. *Science* **253**: 49–53

Hollstein M, Shomer B, Greenblatt M, Soussi T, Hovig E, Montesano R and Harris CC (1996) Somatic point mutations in the *p53* gene of human tumors and cell lines: updated compilation. *Nucleic Acids Res* **24**: 141–146

Horio Y, Suzuki H, Ueda R, Koshikawa T, Sugiura T, Ariyoshi Y, Shimokata K, Takahashi T and Takahashi T (1994) Predominantly tumor-limited expression of a mutant allele in a Japanese family carrying a germline p53 mutation. *Oncogene* **9**: 1231–1235

Iavarone A, Matthay KK, Steinkirchner TM and Israel MA (1992) Germ-line and somatic p53 mutations in multifocal osteogenic sarcoma. *Proc Natl Acad Sci USA* **89**: 4207–4209

Ichii S, Horii A, Nakatsuru S, Furuyama J, Utsunomiya J and Nakamura Y (1992) Inactivation of both *APC* alleles in an early stage of colon adenomas in a patient with familial adenomatous polyposis (FAP). *Hum Mol Genet* **1**: 387–390

Ishioka C, Frebourg T, Yan Y-X, Vidal M, Friend SH, Schmidt S and Iggo R (1993) Screening patients for heterozygous p53 mutations using a functional assay in yeast. *Nature Genet* **5**: 124–129

Jacks T, Remington L, Williams BO, Schmitt EM, Halachmi S, Bronson RT and Weinberg RA (1994) Tumor spectrum analysis in *p53*-deficient mice. *Curr Biol* **4**: 1–7

Jeffrey PD, Gorina S and Pavletich NP (1995) Crystal structure of the tetramerization domain of the p53 tumor suppressor at 1.7 angstroms. *Science* **267**: 1498–1502

Jenkins JR, Rudge K and Currie GA (1984) Cellular immortalization by a cDNA clone encoding the transformation-associated phosphoprotein p53. *Nature* **312**: 651–654

Jenkins JR, Rudge K, Chumakov P and Currie GA (1985) The cellular oncogene p53 can be activated by mutagenesis. *Nature* **317**: 816–818

Jolly KW, Malkin D, Douglass EC, Brown TF, Sinclair AE and Look AT (1994) Splice-site mutation of the p53 gene in a family with hereditary breast-ovarian cancer. *Oncogene* **9**: 97–102

Kastan MB, Onyekwere O, Sidransky D, Vogelstein B and Craig RW (1991) Participation of p53 protein in the cellular response to DNA damage. *Cancer Res* **51**: 6304–6311

Kemp CJ, Wheldon T and Balmain A (1994) p53-deficient mice are extremely susceptible to radiation-induced tumorigenesis. *Nature Genet* **8**: 66–69

Knudson AG, Jr (1971) Mutation and cancer: statistical study of retinoblastoma. *Proc Natl Acad Sci USA* **68**: 820–823

Kovar H, Auinger A, Jug G, Müller T and Pillwein K (1992) p53 mosaicism with an exon 8 germline mutation in the founder of a cancer-prone family. *Oncogene* **7**: 2169–2173

Kuerbitz SJ, Plunkett BS, Walsh WV and Kastan MB (1992) Wild-type p53 is a cell cycle checkpoint determinant following irradiation. *Proc Natl Acad Sci USA* **89**: 7491–7495

Kyritsis AP, Bondy ML, Xiao M, Berman EL, Cunningham JE, Lee PS, Levin VA and Saya H (1994) Germline p53 mutations in subsets of glioma patients. *J Natl Cancer Inst* **86**: 344–349

Lalle P, Moyret-Lalle C, Wang Q, Vialle J-M, Navarro C, Bressac-de Paillerets B, Magaud J-P and Ozturk M (1995) Genomic instability and wild-type p53 function of lymphoblastoid cells with germ-line *p53* mutation. *Oncogene* **10**: 2447–2454

 © Cancer Research Campaign 1997

Lane DP (1992) p53, guardian of the genome. *Nature* **358**: 15–16
Lane DP and Crawford LV (1979) T antigen is bound to a host protein in SV40 transformed cells. *Nature* **278**: 261–263
Lavigueur A, Maltby V, Mock D, Rossant J, Pawson T and Bernstein A (1989) High incidence of lung, bone and lymphoid tumors in transgenic mice overexpressing mutant alleles of the p53 oncogene. *Mol Cell Biol* **9**: 3982–3991
Law JC, Strong LC, Chidambaram A and Ferrell RE (1991) A germ line mutation in exon 5 of the *p53* gene in an extended cancer family. *Cancer Res* **51**: 6385–6387
LeBihan C, Moutou C, Brugières L, Feunteun J and Bonaïti-Pellié C (1995) ARCAD: a method for estimating age-dependent disease risk associated with mutation carrier status from family data. *Genetic Epidemiol* **12**: 13–25
Lee JM and Bernstein A (1993) p53 mutations increase resistance to ionizing radiation. *Proc Natl Acad Sci USA* **90**: 5742–5746
Lee JM, Abrahamson JL, Kandel R, Donehower LA and Bernstein A (1994) Susceptibility to radiation-carcinogenesis and accumulation of chromosomal breakage in p53 deficient mice. *Oncogene* **9**: 3731–3736
Levine AJ, Momand J and Finlay CA (1991) The p53 tumour suppressor gene. *Nature* **351**: 453–456
Li FP and Fraumeni JF (1969*a*) Rhabdomyosarcoma in children; epidemiologic study and identification of a cancer family syndrome. *J Natl Cancer Inst* **43**: 1365–1373
Li FP and Fraumeni JF (1969*b*) Soft-tissue sarcomas, breast cancer and other neoplasms: a familial syndrome? *Ann Int Med* **71**: 747–752
Li FP and Fraumeni JF (1982) Prospective study of a cancer family syndrome. *JAMA* **247**: 2692–2694
Li FP and Fraumeni JF (1994) Collaborative interdisciplinary studies of p53 and other predisposing mutations in Li-Fraumeni syndrome. *Cancer Epidemiology, Biomarkers and Prevention* **3**: 715–717
Li FP, Fraumeni JF, Mulvihill JJ, Blattner WA, Dreyfus MG, Tucker MA and Miller RW (1988) A cancer family syndrome in twenty-four kindreds. *Cancer Res* **48**: 5358–5362
Li FP, Garber JE, Friend SH, Strong LC, Patenaude AF, Juengst ET, Reilly PR, Correa P and Fraumeni JF (1992) Recommendations on predictive testing for germ line p53 mutations among cancer-prone individuals. *J Natl Cancer Inst* **84**: 1156–1160
Li Y-J, Sanson M, Hoang-Xuan K, Delattre J-Y, Poisson M, Thomas G and Hamelin R (1995) Incidence of germ-line *p53* mutations in patients with gliomas. *Int J Cancer* **64**: 383–387
Linzer DIH and Levine AJ (1979) Characterisation of a 54,000 MW cellular SV40 tumor antigen present in SV40-transformed cells and uninfected embryonal carcinoma cells. *Cell* **17**: 43–52
Liu B, Parsons R, Papadopoulos N, Nicolaides NC, Lynch HT, Watson P, Jass JR, Dunlop M, Wyllie A, Peltomaki P, de la Chapelle A, Hamilton SR, Vogelstein B and Kinzler KW (1996) Analysis of mismatch repair genes in hereditary non-polyposis colorectal cancer patients. *Nature Med* **2**: 169–174
Liu PK, Kraus E, Wu TA, Strong LC and Tainsky MA (1996) Analysis of genomic instability in Li-Fraumeni fibroblasts with germline p53 mutations. *Oncogene* **12**: 2267–2278
Livingstone LR, White A, Sprouse J, Livanos E, Jacks T and Tlsty TD (1992) Altered cell cycle arrest and gene amplification potential accompany loss of wild-type p53. *Cell* **70**: 923–935
Lomax ME, Barnes DM, Gilchrist R, Picksley SM, Varley JM and Camplejohn RS (1997) An unusual p53 germline mutation in the oligomerisation domain in a Li–Fraumeni-like family. *Br J Cancer* (in press)
Lubbe J, von Ammon K, Watanabe K, Hegi ME and Kleihues P (1995) Familial brain tumour syndrome associated with a *p53* germline deletion at codon 236. *Brain Pathol* **5**: 15–23
Lustbader ED, Williams WR, Bondy ML, Strom S and Strong LC (1992) Segregation analysis of cancer in families of childhood soft tissue sarcoma patients. *Am J Hum Genet* **51**: 344–356
MacGeoch C, Turner G, Bobrow LG, Barnes DM, Bishop DT and Spurr NK (1995) Heterogeneity in Li-Fraumeni families: p53 mutation analysis and immunohistochemical staining. *J Med Genet* **32**: 186–190
Malkin D, Li FP, Strong LC, Fraumeni JFJ, Nelson CE, Kim DH, Kassel J, Gryka MA, Bischoff FZ, Tainsky MA and Friend SH (1990) Germ line p53 mutations in a familial syndrome of breast cancer, sarcomas, and other neoplasms. *Science* **250**: 1233–1238
Malkin D, Jolly KW, Barbier N, Look AT, Friend SH, Gebhardt MC, Andersen TI, Børresen A-L, Li FP, Garber J and Strong L (1992) Germline mutations of the p53 tumor-suppressor gene in children and young adults with second malignant neoplasms. *N Engl J Med* **326**: 1309–1315
Mazoyer S, Lalle P, Moyret-Lalle C, Marcais C, Schraub S, Frappaz D, Sobol H and Ozturk M (1994) Two germ-line mutations affecting the same nucleotide at codon 257 of p53 gene, a rare site for mutations. *Oncogene* **9**: 1237–1239
McIntyre JF, Smith-Sorensen B, Friend SH, Kassell J, Borresen A-L, Yan YX, Russo C, Sato J, Barbier N, Miser J, Malkin D and Gebhardt MC (1994) Germline mutations of the p53 tumor suppressor gene in children with osteosarcoma. *J Clin Oncol* **12**: 925–930
Metzger AK, Sheffield VC, Duyk G, Daneshvar L, Edwards MSB and Cogen PH (1991) Identification of a germ-line mutation in the p53 gene in a patient with an intracranial ependymoma. *Proc Natl Acad Sci USA* **88**: 7825–7829
Milner J and Metcalf EA (1991) Cotranslation of activated mutant p53 with wild-type drives the wild-type p53 protein into the mutant conformation. *Cell* **65**: 765–774
Nishino H, Knoll A, Buettner VL, Frisk CS, Maruta Y, Haavik J and Sommer SS (1995) p53 wild-type and p53 nullizygous Big Blue transgenic mice have similar frequencies and patterns of observed mutation in liver, spleen and brain. *Oncogene* **11**: 263–270
Parada LF, Land H, Weinberg RA, Wolf D and Rotter V (1984) Cooperation between gene encoding p53 tumour antigen and ras in cellular transformation. *Nature* **312**: 649–651
Parshad R, Price FM, Pirollo KF, Chang EH and Sanford KK (1993) Cytogenetic response to G2-phase X irradiation in relation to DNA repair and radiosensitivity in a cancer-prone family with Li–Fraumeni syndrome. *Radiat Res* **136**: 236–240
Paules RS, Levedakou EN, Wilson SJ, Innes CL, Rhodes N, Tlsty T, Galloway DA, Donehower LA, Tainsky MA and Kaufmann WK (1995) Defective G2 checkpoint function in cells from individuals with familial cancer syndromes. *Cancer Res* **55**: 1763–1773
Pearson ADJ, Craft AW, Ratcliffe JM, Birch JM, Morris Jones PH and Roberts DF (1982) Two families with the Li–Fraumeni cancer family syndrome. *J Med Genet* **19**: 362–365
Plummer SJ, Santibanez-Koref M, Kurosaki T, Liao S, Noble B, Fain PR, Anton-Culver H and Casey G (1994) A germline 2.35kb deletion of p53 genomic DNA creating a specific loss of the oligomerization domain inherited in a Li–Fraumeni syndrome family. *Oncogene* **9**: 3273–3280
Ponder BA (1988) Gene losses in human tumours. *Nature* **335**: 400–402
Porter DE, Holden ST, Steel CM, Cohen BB, Wallace MR and Reid R (1992) A significant proportion of patients with osteosarcoma may belong to Li–Fraumeni cancer families. *J Bone Joint Surg* **74B**: 883–886
Prosser J, Porter D, Coles C, Condie A, Thompson AM, Chetty U, Steel CM and Evans HJ (1992) Constitutional p53 mutation in a non-Li–Fraumeni cancer family. *Br J Cancer* **65**: 527–528
Rogan EM, Bryan TM, Hukku B, Maclean K, Chang AC-M, Moy EL, Englezou A, Warneford SG, Dalla-Pozza L and Reddel RR (1995) Alterations in p53 and $p16^{INK4}$ expression and telomere length during spontaneous immortalisation of Li–Fraumeni syndrome fibroblasts. *Mol Cell Biol* **15**: 4745–4753
Russo CL, McIntyre J, Goorin AM, Link MP, Gebhardt MC and Friend SH (1994) Secondary breast cancer in patients presenting with osteosarcoma: possible involvement of germline p53 mutations. *Med Pediat Oncol* **23**: 354–358
Sah VP, Attardi LD, Mulligan DJ, Williams BO, Bronson RT and Jacks T (1995) A subset of p53-deficient embryos exhibit exencephaly. *Nature Genet* **10**: 175–180
Sameshima Y, Tsunematsu Y, Watanabe S, Tsukamoto T, Kawa-ha K, Hirata Y, Mizoguchi H, Sugimura T, Terada M and Yokota J (1992) Detection of novel germ-line p53 mutations in diverse-cancer-prone families identified by screening patients with childhood adrenocortical carcinoma. *J Natl Cancer Inst* **84**: 703–707
Sands AT, Suroakar MB, Sanchez A, Marth JE, Donehower LA and Bradley A (1995) p53 deficiency does not affect the accumulation of point mutations in a transgenic target. *Proc Natl Acad Sci USA* **92**: 8517–8521
Santibanez-Koref MF, Birch JM, Hartley AL, Jones PH, Craft AW, Eden T, Crowther D, Kelsey AM and Harris M (1991) p53 germline mutations in Li–Fraumeni syndrome. *Lancet* **338**: 1490–1491
Schärer E and Iggo R (1992) Mammalian p53 can function as a transcription factor in yeast. *Nucleic Acids Res* **20**: 1539–1545
Schneider KA, Patenaude AF and Garber JE (1995) Testing for cancer genes: decisions, decisions. *Nature Med* **1**: 942–945
Scott RJ, Krummenacher F, Mary J-L, Weber W, Spycher M and Muller H (1993) Vererbbare p53-mutation bei einem Patienten mit Mehrfachtumoren: Bedeutung fur die genetische Beratung. *Schweiz Med Wochenschr* **123**: 1287–1292
Shay JW, Tomlinson G, Piatyszek MA and Gollahon LS (1995) Spontaneous in vitro immortalization of breast epithelial cells from a patient with Li–Fraumeni syndrome. *Mol Cell Biol* **15**: 425–432

© Cancer Research Campaign 1997

Shi Y-P, Venkatachalam S, Jones S, Vogel H, Bradley A, Pinkel D and Donehower LA (1997) Retention of the wild-type allele in tumors from p53-deficient heterozygous mice: is p53 an exception to the two hit hypothesis? *Nature Genet* (submitted)

Shiseki M, Nishikawa R, Yamamoto H, Ochiai A, Sugimura H, Shitara N, Sameshima Y, Mizoguchi H, Sugimura T and Yokota J (1993) Germ-line *p53* mutation is uncommon in patients with triple primary cancers. *Cancer Lett* **73**: 51–57

Sidransky D, Tokino T, Helzlsouer K, Zehnbauer B, Rausch G, Shelton B, Prestigiacomo L, Vogelstein B and Davidson N (1992) Inherited *p53* gene mutations in breast cancer. *Cancer Res* **52**: 2984–2986

Smith SA, Easton DF, Evans DGR and Ponder BAJ (1992) Allele losses in the region 17q12-21 in familial breast and ovarian cancer involve the wild-type chromosome. *Nature Genet* **2**: 128–131

Soussi T, Caron de Fromentel C and May P (1990) Structural aspects of the p53 protein in relation to gene evolution. *Oncogene* **5**: 945–952

Speiser P, Gharehbaghi-Schnell E, Eder S, Haid A, Kovarik J, Nenutil R, Sauter G, Schneeberger CH, Vojtesek B, Wiltschke CH and Zeillinger R (1996) A constitutional *de novo* mutation in exon 8 of the p53 gene in a patient with multiple primary malignancies. *Br J Cancer* **74**: 269–273

Sproston ARM, Boyle JM, Heighway J, Birch JM and Scott D (1996) Fibroblasts from Li–Fraumeni patients are resistant to low dose-rate irradiation. *Int J Radiat Biol* **70**: 145–150

Srivastava S, Zou Z, Pirollo K, Blattner W and Chang EH (1990) Germ-line transmission of a mutated p53 gene in a cancer-prone family with Li–Fraumeni syndrome. *Nature* **348**: 747–749

Srivastava S, Tong YA, Devadas K, Zou Z-Q, Sykes VW, Chen Y, Blattner WA, Pirollo K and Chang EH (1992) Detection of both mutant and wild-type p53 protein in normal skin fibroblasts and demonstration of a shared 'second hit' on p53 in diverse tumors from a cancer-prone family with Li–Fraumeni syndrome. *Oncogene* **7**: 987–991

Stewart N, Hicks GG, Paraskevas F and Mowat M (1995) Evidence for a second cell cycle block at G2/M by p53. *Oncogene* **10**: 109–115

Stolzenberg M-C, Brugières L, Gardes M, Dessarps-Freichey F, Chompret A, Bressac B, Lenoir G, Bonaïti-Pellié C, Lemerle J and Feunteun J (1994) Germ-line exclusion of a single p53 allele by premature termination of translation in a Li–Fraumeni family. *Oncogene* **9**: 2799–2804

Strauss EA, Hosler MR, Herzog P, Salhany K, Louie R and Felix CA (1995) Complex replication error causes *p53* mutation in a Li–Fraumeni family. *Cancer Res* **55**: 3237–3241

Strong LC and Williams WR (1987) The genetic implications of long-term survival of childhood cancer. *Am J Pediatr Hematol Oncol* **9**: 99–103

Strong LC, Stine M and Norsted TL (1987) Cancer in survivors of childhood soft tissue sarcoma and their relatives. *J Natl Cancer Inst* **79**: 1213–1220

Sun X-F, Johannsson O, Hakansson S, Sellberg G, Nordenskjold B, Olsson H and Borg A (1996) A novel *p53* germline alteration identified in a late onset breast cancer kindred. *Oncogene* **13**: 407–411

Toguchida J, Yamaguchi T, Dayton SH, Beauchamp RL, Herrera GE, Ishizaki K, Yamamuro T, Meyers PA, Little JB, Sasaki MS, Weichselbaum RR and Yandell DW (1992) Prevalence and spectrum of germline mutations of the p53 gene among patients with sarcoma. *N Engl J Med* **326**: 1301–1308

Tory K, Brauch H, Linehan M, Barba D, Oldfield E, Filling-Katz M, Seizinger B, Nakamura Y, White R, Marshall FF, Lerman MI and Zbar B (1989) Specific genetic change in tumors associated with von Hippel–Lindau disease. *J Natl Cancer Inst* **81**: 1097–1101

Tsutsui T, Fujino T, Kodama S, Tainsky MA, Boyd J and Barrett JC (1995) Alfatoxin B1-induced immortalization of cultured skin fibroblasts from a patient with Li–Fraumeni syndrome. *Carcinogenesis* **16**: 25–34

Varley JM, McGown G, Thorncroft M, Tricker KJ, Teare MD, Santibanez-Koref MF, Houlston RS, Martin J, Birch JM and Evans DGR (1995) An extended Li–Fraumeni kindred with gastric carcinoma and a codon 175 mutation in TP53. *J Med Genet* **32**: 946–950

Varley JM, Thorncroft M, McGown G, Tricker K, Birch JM and Evans DGR (1996*a*) A novel deletion within exon 6 of *TP53* in a family with Li Fraumeni-like syndrome, and LOH in a benign lesion from a mutation carrier. *Cancer Genet Cytogenet* **90**: 14–16

Varley JM, McGown G, Thorncroft M, Cochrane S, Morrison P, Woll P, Kelsey AM, Mitchell ELD, Boyle J, Birch JM and Evans DGR (1996*b*) A previously undescribed mutation within the tetramerisation domain of *TP53* in a family with Li–Fraumeni syndrome. *Oncogene* **12**: 2437–2442

Varley JM, McGown G, Thorncroft M, Santibanez-Koref MF, Kelsey AM, Tricker KM, Evans DGR and Birch JM (1997*a*) Germline mutations of TP53 in Li–Fraumeni families: an extended study of 39 families. (submitted)

Varley JM, Thorncroft M, McGown G, Appleby J, Kelsey AM, Tricker KM, Evans DGR and Birch JM (1997*b*) A detailed study of loss of heterozygosity on chromosome 17 in tumours from Li–Fraumeni patients carrying a mutation to the *TP53* gene. *Oncogene* **14**: 865–871

Vogelstein B (1990) A deadly inheritance. *Nature* **348**: 681–682

Vogelstein B and Kinzler KW (1994) X-rays strike p53 again. *Nature* **370**: 174–175

Wagner J, Portwine C, Rabin K, Leclerc J-M, Narod SA and Malkin D (1994) High frequency of germline p53 mutations in childhood adrenocortical cancer. *J Natl Cancer Inst* **86**: 1707–1710

Warneford SG, Witton LJ, Townsend ML, Rowe PB, Reddel RR, Dalla-Pozza L and Symonds G (1992) Germ-line splicing mutation of the *p53* gene in a cancer-prone family. *Cell Growth Different* **3**: 839–846

Williams KJ, Heighway J, Birch JM, Norton JD and Scott D (1996) No defect in $G_1$/S cell cycle arrest in irradiated Li–Fraumeni lymphoblastoid cell lines. *Br J Cancer* **74**: 698–703

Williams KJ, Boyle JM, Birch JM, Norton JD and Scott D (1997) Cell cycle arrest defect in Li–Fraumeni syndrome: a mechanism of cancer predisposition? *Oncogene* **14**: 277–282

Yin Y, Tainsky MA, Bischoff FZ, Strong LC and Wahl GM (1992) Wild-type p53 restores cell cycle control and inhibits gene amplification in cells with mutant p53 alleles. *Cell* **70**: 937–948

Yonish-Rouach E, Resnitzky D, Lotem J, Sachs L, Kimchi A and Oren M (1991) Wild-type p53 induces apoptosis of myeloid leukaemic cells that is inhibited by interleukin-6. *Nature* **352**: 345–347

© Cancer Research Campaign 1997